# Association of Glutamate Infusion With Risk of Acute Kidney Injury After Coronary Artery Bypass Surgery: A Pooled Analysis of 2 Randomized Clinical Trials

**DOI:** 10.1001/jamanetworkopen.2023.51743

**Published:** 2024-01-22

**Authors:** Jonas Holm, Farkas Vanky, Rolf Svedjeholm

**Affiliations:** 1 Department of Thoracic and Vascular Surgery, Department of Health, Medicine and Caring Sciences, Unit of Cardiovascular Medicine, Linköping University, Linköping, Sweden

## Abstract

**Importance:**

Acute kidney injury (AKI) after cardiac surgery is associated with increased morbidity and mortality, and measures to prevent AKI have had limited success. Glutamate has been reported to enhance natural postischemic recovery of the heart, but not among animals and humans with diabetes.

**Objective:**

To summarize pooled results from the GLUTAMICS (Glutamate for Metabolic Intervention in Coronary Surgery) trials regarding the effect of glutamate on postoperative AKI among patients without diabetes undergoing coronary artery bypass graft (CABG) surgery.

**Design, Setting, and Participants:**

Data on a total of 791 patients without diabetes from 2 prospective, randomized, double-blind multicenter trials performed at 5 cardiac surgery centers in Sweden between October 4, 2005, and November 12, 2009, and between November 15, 2015, and September 30, 2020, were pooled. Patients had acute coronary syndrome, left ventricular ejection fraction of 0.30 or less, or a European System for Cardiac Risk Evaluation II score of 3.0 or more and underwent CABG with or without additional valve procedure. Statistical analysis was performed from May to November 2023.

**Interventions:**

Intravenous infusion of 0.125-M l-glutamic acid or saline at 1.65 mL/kg/h for 2 hours during reperfusion, after which the infusion rate was halved and an additional 50 mL was given.

**Main Outcomes and Measures:**

The primary end point was AKI, defined as postoperative increase of plasma creatinine of 50% or more, corresponding to the Risk stage or higher in the Risk, Injury, Failure, Loss, and End-Stage kidney disease (RIFLE) criteria.

**Results:**

A total of 791 patients without diabetes (391 who received glutamate [mean (SD) age, 69.3 (9.1) years; 62 women (15.9%)] and 400 controls [mean (SD) age, 69.6 (9.5) years; 73 women (18.3%)]) were randomized. Baseline data did not differ between groups. Glutamate was associated with a significantly lower risk of AKI (relative risk, 0.49 [95% CI, 0.29-0.83]). Dialysis was required for 2 patients in the glutamate group and 5 patients in the control group. In multivariable analysis, glutamate remained significantly associated with a protective effect against AKI (odds ratio, 0.47 [95% CI, 0.26-0.86]). In the glutamate and control groups, the rate of postoperative mortality at 30 days or less was 0.5% (2 of 391) vs 1.0% (4 of 400), and the rate of stroke at 24 hours or less was 0.8% (3 of 391) vs 1.8% (7 of 400).

**Conclusions and Relevance:**

In this pooled analysis of 2 randomized clinical trials, infusion of glutamate was associated with a markedly lower risk of AKI after CABG among patients without diabetes. The findings are exploratory and need to be confirmed in prospective trials.

**Trial Registration:**

ClinicalTrials.gov Identifiers: NCT00489827 and NCT02592824

## Key Points

**Question:**

Can intravenous glutamate infusion prevent acute kidney injury (AKI) among patients without diabetes undergoing coronary artery bypass surgery?

**Findings:**

In a pooled analysis of 2 randomized clinical trials with a total of 791 patients, glutamate infusion was associated with a significantly lower incidence of AKI (defined as a postoperative increase of plasma creatinine ≥50%) among patients without diabetes.

**Meaning:**

As previous measures to treat or prevent postoperative AKI have had limited success, further studies of intravenous glutamate infusion are warranted to see if the results can be reproduced and confirmed.

## Teaser

This pooled analysis of 2 randomized clinical trials summarizes pooled results from the GLUTAMICS trials regarding the effect of glutamate on postoperative acute kidney injury (AKI) among patients without diabetes undergoing coronary artery bypass graft surgery.

## Introduction

Acute kidney injury (AKI) is a common complication in cardiac surgery associated with increased postoperative morbidity and mortality.^1,2,3,4^ Even moderate impairment of kidney function carries significant prognostic implications.^5,6^ Many causes and risk factors of AKI have been described, but measures to prevent or treat kidney injury have had limited success.^1,2,7^ Maintenance of an adequate hemodynamic state and kidney perfusion is essential during cardiopulmonary bypass (CPB) and after surgery.^1,2,8^ However, sufficient evidence for a preventive effect of pharmacologic drugs on AKI has not been achieved.^1,2,7^

Glutamate plays a key role in myocardial metabolism during ischemia and reperfusion.^9,10,11,12,13,14^ The myocardial glutamate and aspartate pool is consumed during ischemia.^15^ In animal experiments, glutamate administration facilitates postischemic recovery of myocardial metabolism and contractile function.^10,11,12,13^ In humans, the heart adapts to ischemia by increasing consumption of glutamate and the release of alanine.^16,17^ High fractional extraction rates of glutamate by the heart early after coronary artery bypass graft (CABG) surgery preceded recovery of normal myocardial metabolism.^18^ However, myocardial uptake was limited by plasma content of glutamate. Intravenous infusion of glutamate during reperfusion increased myocardial uptake of glutamate and was associated with improved myocardial metabolism and cardiac function.^19,20^

The Glutamate for Metabolic Intervention in Coronary Surgery (GLUTAMICS) trials investigated whether intravenous glutamate infusion can prevent postoperative myocardial dysfunction after CABG.^21,22^ Both trials had negative results, largely due to shifts in the population submitted by cardiologists for CABG. The first trial was initially intended for patients with unstable Canadian Cardiovascular Society (CCS) class IV angina but, due to shifts in cardiologic practice, a large proportion of patients submitted for urgent surgery were low-risk patients with non–ST-elevation myocardial infarction. Among patients with CCS class IV angina, a significant reduction in the incidence of severe postoperative heart failure was found. In the second trial, the proportion of patients with diabetes, a cohort reported to have a blunted effect of glutamate, had doubled to 47%.^21,22,23,24^ The mechanism appears to be related to downregulation of mitochondrial glutamate transporter EAAT1 (excitatory amino acid transporter 1) in the heart of humans and animals with diabetes.^23,24^

Although both GLUTAMICS trials had negative results, secondary analyses suggested that further evaluation was warranted due to findings supporting the assumption that glutamate prevents postoperative heart failure in patients without diabetes. In addition, the incidence of AKI, defined as AKI Risk, Injury, Failure, Loss, and End-stage kidney disease (RIFLE) criteria stage I or higher, was lower among patients without diabetes who received glutamate. However, the sample size was small and there were differences in baseline data.^22^ Therefore, we decided to revisit both trials to address the effect of glutamate infusion on postoperative AKI among all patients studied so far.

## Methods

### Study Design

This study summarizes the pooled results of all patients without diabetes undergoing CABG with or without additional valve using CPB in the GLUTAMICS I and II trials between October 4, 2005, and November 12, 2009, and between November 15, 2015, and September 30, 2020 (CONSORT diagram in the Figure).^21,22^ These investigator-initiated trials were multicenter and designed as prospective, externally randomized, placebo-controlled, double-blind trials with parallel assignment to glutamate or placebo (saline). The trials were approved by the Swedish Medical Products Agency and registered at ClinicalTrials.gov (NCT00489827 and NCT02592824; trial protocols in Supplement 1). The Regional Ethical Review Board in Linköping approved the trials and their amendments. All patients were enrolled after providing written informed consent. The studies were performed in accordance with the Declaration of Helsinki,^25^ and we followed the Consolidated Standards of Reporting Trials (CONSORT) reporting guideline.

**Figure.**
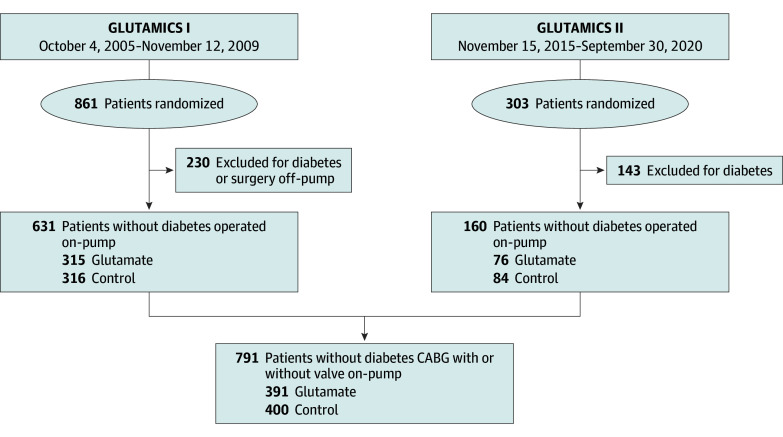
CONSORT Diagram CONSORT flowchart of how patients were selected from the GLUTAMICS (Glutamate for Metabolic Intervention in Coronary Surgery) I and GLUTAMICS II trials. GLUTAMICS I comprised patients undergoing coronary artery bypass graft (CABG) surgery with or without valve for acute coronary syndrome; GLUTAMICS II comprised patients undergoing CABG with or without valve on-pump for patients with left ventricular ejection fraction of 0.30 or less or European System for Cardiac Operative Risk Evaluation II score of 3.0 or more with cardiac risk factor. Flowcharts for the GLUTAMICS I and GLUTAMICS II trials have been given in respective publications.^21,22^ Off-pump indicates CABG without cardiopulmonary bypass; and on-pump, CABG with use of cardiopulmonary bypass.

### Study Population

We identified 791 patients from the GLUTAMICS I and II trials from 5 cardiac surgery centers in Sweden (Linköping, Örebro, Karlskrona, Umeå, and Gothenburg) without a diagnosis of diabetes. Patients had been accepted for CABG with CPB due to at least 2-vessel disease or left main stenosis, with or without a concomitant procedure. Patients from the first trial had been included because they underwent urgent procedures due to acute coronary syndrome. Patients from the GLUTAMICS II trial carried a moderate to high risk of developing postoperative heart failure due to a severely reduced preoperative left ventricular ejection fraction of 0.30 or less or a European System for Cardiac Risk Evaluation II score of 3.0 or more, with at least 1 of the following cardiac or procedure-related risk factors: an urgent or emergency procedure, CCS class IV angina, left ventricular ejection fraction of 0.50 or less, myocardial infarction within 90 days prior, or a concomitant aortic or mitral valve procedure (Figure).^21,22^

Exclusion criteria were age older than 85 years; ambiguous food allergy known to have triggered shortness of breath, headache, or flushing; previous cardiac surgery; informed consent not possible due to a critical condition or other reason; preoperative use of mechanical circulatory assistance; kidney failure with preoperative dialysis or calculated creatinine clearance less than 30 mL/min; surgery without heart-lung machine; concomitant Maze procedure; surgery on the ascending aorta; and surgery on both the aortic and mitral valves. Both trials underwent professional external monitoring.^21,22^ Due to funding, the degree of monitoring differed between the 2 trials, but for all patients, it confirmed the correct inclusion, provision of informed written consent, primary and secondary end points, and records of adverse events.

### Interventions

Patients were randomly allocated in a 1:1 ratio to a blinded intravenous infusion of either a 0.125-M l-glutamic acid solution or saline at a rate of 1.65 mL/kg/h. The infusion was given once in association with surgery. The infusion protocols differed slightly but in both trials the solution was infused for 2 hours after release of aortic cross-clamp. After that, the infusion rate was halved, with an additional 50 mL given.^21,22^ The maximum volume infused was 500 mL.

The dosage of glutamate was based on studies addressing arterial levels and myocardial extraction of glutamate at different infusion rates.^26^ The duration of treatment and the practice to halve the infusion rate for the last 50 mL was based on clinical experience with glutamate infusion regarding hemodynamic recovery in patients at high risk.^27^

### Glutamate Solution

For the glutamate solution, 500 mL of a 0.125-M solution of l-glutamic acid with pH 6.0 and 280 mOsm/kg contained 9.2 g of l-glutamic acid, 0.8 g of NaCl, H_2_O up to 500 mL, and NaOH as needed. The production of glutamate solution was done by Apoteket Produktion & Laboratorier.^28^

### Outcomes and Safety End Points

The primary end point used for this study was the incidence of postoperative increase of plasma creatinine by 50% or more, corresponding to RIFLE stage Risk or higher. Plasma creatinine was measured preoperatively and on postoperative days 1 and 3 for all patients and whenever clinical course motivated it. Plasma creatinine was measured daily according to clinical routine for patients with an extended stay in the intensive care unit (ICU). The main safety end points were similar in both trials and included postoperative stroke within 24 hours, postoperative mortality within 30 days, and suspected unexpected serious adverse reactions within 24 hours.

### Sample Size, Randomization, and Blinding

Sample size was limited by the available number of patients without diabetes in the 2 GLUTAMICS trials. Randomization was computer generated by the producer of the study solutions (Apoteket Produktion & Laboratorier) in variable block sizes. Randomization was stratified for isolated CABG and CABG with additional valve procedure in the second trial. Patients, staff, and investigators were blinded to the infused treatment (clear transparent solutions).

### Clinical Management

Clinical management was standardized and similar at the participating centers, with minor differences. Cardiopulmonary bypass and aortic cross-clamping were used. Cold blood cardioplegia was used for myocardial protection. The CPB was performed using a nonpulsatile technique, in normothermia (35.5-36.5 °C), with a blood flow index of 2.4 L/m^2^. Perfusion pressure was adjusted to maintain continuous urine output. Inotropes were given at the discretion of the attending physician. Intraoperative and postoperative glycemic control were used at all participating centers, with a plasma glucose target of 90 to 180 mg/dL (to convert glucose to millimoles per liter, multiply by 0.0555). Insulin infusion was started at a plasma glucose level of 144 mg/dL (Örebro) or 180 mg/dL (other centers).

### Definitions

Diabetes was defined as patients with a diagnosis of type 1 or 2 diabetes on admission to the cardiac surgical department. Severe left ventricular dysfunction corresponds to an ejection fraction of 0.30 or less. Postoperative mortality was defined as mortality within 30 days of surgery.

Postoperative stroke was defined as neurologic or cognitive deficit with a cerebral injury verified on computed tomography (CT) scan. All patients with suspected stroke underwent computed tomography scan. Stroke within 24 hours of surgery was defined as a stroke that occurred within 24 hours of surgery, or signs of a stroke when first assessable in deeply sedated patients receiving mechanical ventilation. Acute kidney injury was defined according to RIFLE criteria based on plasma creatinine alone as a postoperative increase in plasma creatinine of at least 50%.^6^ Estimated glomerular filtration rate or urine output criteria were not used.

Postoperative myocardial injury was measured with creatine kinase MB isoenzyme on the first postoperative morning. Different troponin analyses were used at the participating sites and, hence, are not presented.

### Statistical Analysis

Statistical analysis was performed from May to November 2023. A 2-sided *t* test or Mann-Whitney test was used for between-group comparisons of continuous variables. The Levene test was used to assess equality of variances. Categorical variables, including the primary and safety end points, were analyzed with the Fisher exact test. Depending on the test used, results are presented as mean (SD) values, median (IQR) values, or numbers and percentages. Exploratory analyses were done with multiple logistic regression. To address confounders, all preoperative and intraoperative variables in Table 1 and Table 2, site, and trial were tested in the model. Statistical significance was defined as *P* < .05. Statistical analyses were performed with Statistica, version 13.5.0.17 (TIBCO Software Inc) and IBM SPSS, version 29.0.0.0.0 (IBM Corp).

**Table 1.** Preoperative Characteristics of the Glutamate and Control Groups

Variable	Glutamate (n = 391)	Control (n = 400)
Age, mean (SD), y	69.3 (9.1)	69.6 (9.5)
Female sex, No. (%)	62 (15.9)	73 (18.3)
BMI, mean (SD)	26.6 (3.9)	27.0 (4.2)
EuroSCORE I score, mean (SD), %	5.1 (2.7)^a^	4.9 (2.7)^b^
EuroSCORE II score, mean (SD), %	3.6 (2.2)^c^	3.7 (2.6)^d^
Hypertension, No./total No. (%)	208/383 (54.3)	234/399 (58.6)
COPD, No./total No. (%)	35/387 (9.0)	28/396 (7.1)
Peripheral arterial disease, No./total No. (%)	48/378 (12.7)	46/391 (11.8)
Cerebrovascular disease, No./total No. (%)	24/387 (6.2)	34/400 (8.5)
Plasma creatinine, mean (SD), mg/dL	1.1 (0.3)	1.1 (0.3)
Estimated CrCl, mean (SD), mL/min	75.1 (25.5)	75.0 (27.6)
NT-proBNP, mean (SD), ng/L	1406 (2447)^e^	1320 (1652)^e^
Left main stenosis, No./total No. (%)	164/390 (41.8)	143/399 (35.8)
AMI ≤3 wk before surgery, No./total No. (%)	232/390 (59.5)	230/400 (57.5)
CCS class IV angina, No. (%)	183 (46.8)	188 (47.0)
Atrial fibrillation, No./total No. (%)	33/389 (8.5)	40/396 (10.1)
Severe LV dysfunction, No./total No. (%)	37/390 (9.5)	34/399 (8.5)

Abbreviations: AMI, acute myocardial infarction; BMI, body mass index (calculated as weight in kilograms divided by height in meters squared); CCS, Canadian Cardiovascular Society; COPD, chronic obstructive pulmonary disease; CrCl, creatinine clearance according to the Cockcroft-Gault formula; EuroSCORE, European System for Cardiac Operative Risk Evaluation; LV, left ventricular; NT-proBNP, N-terminal pro–brain natriuretic peptide.

SI conversion factor: To convert plasma creatinine to micromoles per liter, multiply by 88.4.

^a^ For 315 patients.

^b^ For 316 patients.

^c^ For 229 patients.

^d^ For 233 patients.

^e^ For 223 patients.

**Table 2.** Intraoperative and Postoperative Characteristics of the Glutamate Group and the Control Group (Saline)

Variable	Glutamate (n = 391)	Control (n = 400)	*P* value
Urgent or emergency procedure, No. (%)	355 (90.8)	363 (90.8)	>.99
Number of bypasses, mean (SD)	3.8 (1.1)	3.7 (1.1)	.18
Additional valve procedure, No. (%)	35 (9.0)	39 (9.8)	.72
Aortic cross-clamp time, mean (SD), min	56 (24)	57 (27)	.50
CPB time, mean (SD), min	88 (35)	89 (37)	.76
NT-proBNP on postoperative day 3, mean (SD), ng/L	5739 (4590)^a^	6680 (5929)^b^	.07
Increase of NT-proBNP on postoperative day 3, mean (SD), ng/L	4287 (4434)^c^	5343 (5188)^d^	.03
CK-MB on postoperative day 1, median [IQR], ng/mL	14 (10-25)^e^	15 (10-23)^f^	.97
ICU stay, median (IQR), d	0.9 (0.7-1.0)	0.9 (0.7-1.0)	.54
Mechanical ventilation time, median (IQR), h	4.2 (2.7-6.2)	4.4 (2.9-6.7)	.12
Mechanical ventilation time >48 h, No./total No. (%)	13/390 (3.3)	14/400 (3.5)	>.99
IABP, No. (%)	1 (0.3)	5 (1.2)	.22
Reoperation bleeding, No. (%)	23 (5.9)	26 (6.5)	.77
Postoperative atrial fibrillation, No. (%)	148 (37.9)	169 (40.0)	.56
Postoperative stroke ≤24 h, No./total No. (%)	3/391 (0.8)	7/400 (1.8)	.34
Increase in plasma creatinine, mean (SD), mg/dL	0.1 (0.4)^g^	0.2 (0.5)	.11
RIFLE stage ≥Risk, No./total No. (%)	19/390 (4.9)	40/400 (10.0)	.007
RIFLE stage ≥Injury, No./total No. (%)	4/390 (1.0)	16/400 (4.0)	.01
Dialysis, No. (%)	2 (0.5)	5 (1.2)	.45
Mortality ≤30 d, No. (%)	2 (0.5)	4 (1.0)	.69

Abbreviations: CK-MB, creatine kinase-MB isoenzyme; CPB, cardiopulmonary bypass; IABP, intra-aortic balloon pump; ICU, intensive care unit; NT-proBNP, N-terminal pro-brain natriuretic peptide; RIFLE, Risk, Injury, Failure, Loss, and End-stage kidney disease criteria.

SI conversion factors: To covert CK-MB to micrograms per liter, multiply by 1.0; and plasma creatinine to micromoles per liter, multiply by 88.4.

^a^ For 202 patients.

^b^ For 210 patients.

^c^ For 197 patients.

^d^ For 204 patients.

^e^ For 365 patients.

^f^ For 383 patients.

^g^ For 390 patients.

## Results

### Patient Characteristics

A total of 791 patients without diabetes (391 who received glutamate [mean (SD) age, 69.3 (9.1) years; 62 women (15.9%)] and 400 controls [mean (SD) age, 69.6 [9.5] years; 73 women (18.3%)]) were randomized (Table 1). Complete data on plasma creatinine was available in 790 patients. Baseline data did not differ significantly between groups, and preoperative kidney function was almost similar in both groups.

### Postoperative Kidney Function

Glutamate was associated with a lower incidence of postoperative increase of plasma creatinine of 50% or more, corresponding to AKI RIFLE stage Risk or higher (relative risk [RR], 0.49 [95% CI, 0.29-0.83]; *P* = .008). The incidence of postoperative increase of plasma creatinine of 100% or more, corresponding to AKI RIFLE stage Injury, was also lower in the glutamate group (RR, 0.26 [95% CI, 0.09-0.76]; *P* = .01). Dialysis was required for 2 patients in the glutamate group and 5 patients in the control group. Details are given in Table 2.

In multivariable analysis, glutamate remained significantly associated with a protective effect against AKI (odds ratio, 0.47 [95% CI, 0.26-0.86]; *P* = .02). Risk factors in the model were increasing age, increasing body mass index, time on CPB, additional aortic valve procedure, and preoperative left ventricular ejection fraction of 0.30 or less (Table 3).

**Table 3.** Multivariable Logistic Regression Results for Preoperative and Intraoperative Variables Significantly Associated With Postoperative AKI Among Patients Without Diabetes^a^

Variable	*B* (SE)	OR (95% CI)	*P* value
Age, y	0.080 (0.022)	1.08 (1.04 to 1.13)	<.001
BMI	0.135 (0.034)	1.14 (1.07 to 1.22)	<.001
CPB time, min	0.011 (0.004)	1.01 (1.00 to 1.02)	.004
Glutamate	−0.750 (0.308)	0.47 (0.26 to 0.86)	.02
CABG plus AVR	1.061 (0.444)	2.89 (1.21 to 6.89)	.02
LVEF ≤0.30	0.883 (0.399)	2.42 (1.11 to 5.28)	.03

Abbreviations: AKI, acute kidney injury (defined as Risk, Injury, Failure, Loss, and End-stage kidney disease criteria stage of Risk or higher); AVR, aortic valve replacement; BMI, body mass index; CABG, coronary artery bypass grafting; CPB, cardiopulmonary bypass; LVEF, left ventricular ejection fraction; OR, odds ratio.

^a^ Multivariable logistic regression model. Nagelkerke (*R*^2^ = 0.220); Hosmer-Lemeshow goodness-of-fit test (χ^2^_8_ = 8.930; *P* = .35). According to receiver operating characteristic analysis, the area under the curve for the model was 0.82 (95% CI, 0.78-0.86). The area under the curve for glutamate alone was 0.59 (95% CI, 0.52-0.67).

The second trial was designed to include only high-risk patients and the effect of glutamate was more pronounced in that trial than the first trial (RR, 0.37 [95% CI, 0.18-0.77] vs RR, 0.69 [95% CI, 0.33-1.47]). Adjusting for trial in the multivariable analysis would have had only a minimal effect on the role of glutamate (OR, 0.48 [95% CI, 0.26-0.87]; *P* = .02).

The effect of study site was also tested in the multivariable analysis and found to be negligible. Three of the sites included few patients and data for these sites were pooled. The RR per site was 0.37 (95% CI, 0.14-0.99) in Örebro, 0.55 (95% CI, 0.28-1.09) in Linköping, and 0.50 (95% CI, 0.10-2.59) in pooled sites.

### Safety Outcomes

The rate of postoperative mortality within 30 days was 0.5% (2 of 391) vs 1.0% (4 of 400) in the glutamate group and control group, respectively, and the rate of stroke within 24 hours was 0.8% (3 of 390) vs 1.8% (7 of 400) (Table 2). No adverse events related to glutamate were observed.

## Discussion

This study reports the pooled results of all available data on postoperative increase of plasma creatinine after CABG among patients without diabetes randomized to glutamate or saline. Intravenous glutamate infusion was associated with a significantly lower incidence of postoperative AKI among patients without diabetes undergoing CABG. Multivariable analysis identified glutamate infusion as the only factor associated with a reduced risk of AKI.

The results agree with findings from GLUTAMICS II in a much smaller sample with baseline differences.^22^ Some of the patients do overlap, but the sample size of this study is almost 5 times larger. Furthermore, baseline data do not differ significantly and are almost similar regarding important risk factors, such as age and preoperative kidney function.

All in all, this exploratory analysis suggests that intravenous glutamate infusion may provide an effective means of preventing postoperative kidney dysfunction. This finding is important because we currently lack effective preventive treatment and because even moderate kidney dysfunction carries significant prognostic implications.^1,2,5,6^

A potential mechanism behind a renoprotective effect of intravenous glutamate infusion is improved recovery of cardiac function after ischemia. This possibility is supported by animal and human experimental data suggesting improved recovery of oxidative metabolism and contractile function by replenishment of Krebs cycle intermediates.^10,11,12,13,19,20^ The first GLUTAMICS trial included a high proportion of patients at low risk, but among high-risk patients, glutamate was associated with a lower incidence of severe postoperative heart failure leading to extended ICU stay or death. Patients who fulfilled prespecified criteria for heart failure at weaning from CPB demonstrated improved recovery with shorter ICU stay and less need for inotropes if they were treated with glutamate.^21,29^ Clinical experience agrees with these findings.^27,30^ In GLUTAMICS II, glutamate infusion was associated with mitigated postoperative increases of N-terminal pro-brain natriuretic peptide (NT-proBNP) and plasma copeptin in patients without diabetes.^22,31^ However, these biomarkers for heart failure also increase in association with kidney dysfunction, which complicates the interpretation.^32,33^

There are no data to support a direct renoprotective effect of glutamate. On the contrary, studies using high doses of glutamate in murine models suggest that glutamate signaling may play a role in the pathogenesis of AKI.^34,35^ However, nephrotoxic effects caused by high doses of glutamate in rodents probably has little relevance for the doses used in humans.^36^ Glutamate is one of the few amino acids extracted by the heart early after CABG, and, in fact, the one most abundantly extracted by heart and skeletal muscle under these conditions.^18,37^ Glutamate uptake in skeletal muscle correlates with glutamine release.^37^ Thus, glutamate could through transamination provide an indirect source of glutamine, an amino acid potentially beneficial to the kidneys, intestine, and immune system.^26,38,39,40^

The fact that the mean increase of plasma creatinine did not reach statistical significance suggests an effect in high-risk patients rather than a general effect in all patients. This finding speaks in favor of an effect related to improved recovery of cardiac function as the main cause for the reduced incidence of AKI. Some degree of myocardial metabolic derangement is required for glutamate to make a difference regarding recovery of myocardial function.^19,20,41^

In clinical practice we have used glutamate infusion as part of a metabolic strategy for more than 2 decades, initially for treatment of critically ill patients and due to encouraging experience also for prevention among patients at high risk.^27,30,42^ In line with the current results, preservation of kidney function with glutamate has been encouraging.^27,42^

No adverse effects directly attributable to glutamate have been detected in our clinical experience or in either of the 2 trials.^21,22^ Safety outcomes, such as postoperative mortality and stroke, did not differ. Potential neurotoxic effects related to glutamate have been suggested based on findings in rodents receiving high doses of glutamate.^43^ As an additional precautionary step, a study on the S100B protein to detect potential subclinical neurologic injury was conducted and did not raise any concerns.^44^

### Limitations and Strengths

This study has some limitations. The main limitation of this report is that it is a retrospective analysis. Postoperative kidney function was not a prespecified end point, and only 2 trials, conducted by the same investigators, were available for analysis. The criteria for AKI were based on plasma creatinine alone. Urine output was not accounted for, as data were not recorded in the database. Measurement of urine output allows for earlier detection of AKI and is associated with a higher incidence of AKI contributing to heterogeneity in the literature, while the added value of diuresis for assessment of kidney injury remains unclear.^45^

Other markers for kidney function that are more sensitive and specific could have been considered but, on the other hand, this study reflects how kidney failure is assessed in clinical practice at many centers. Sampling for plasma creatinine was conducted according to protocols intended for other purposes and may in some cases have missed the peak value. However, patients with a complicated postoperative course had daily checks of plasma creatinine according to clinical routine.

There were some differences between the 2 trials.^21,22^ The glutamate infusion was started at different time points in each trial but in both studies the infusion was continued for 150 minutes after release of the aortic cross-clamp, with the infusion rate halved during the last 30 minutes.

The first trial included a high proportion of patients at low risk, whereas the second trial was designed to include high-risk patients,^21,22^ which probably explains why the relative risk reduction associated with glutamate was more pronounced in the second trial. However, the proportion of patients from both trials was similar in the study groups and glutamate remained significantly associated with a lower risk of AKI after adjusting for trial, site, and significant risk factors. The aim of these analyses was to exclude that the results were explained by confounders but, nevertheless, it is essential to emphasize that the results are exploratory and should only be regarded as hypothesis generating.

This study also has some strengths, including that the patient cohorts were evenly balanced and that it included all patients without diabetes undergoing CABG studied so far with this objective. Furthermore, the results are not explained by a poor outcome in the control group, as the reported incidence of AKI in the literature ranges between 7% and 40%.^3^ Possible reasons for the favorable results in general could be the omission of patients with diabetes and that the sickest patients treated on an emergency basis were not included due to ethical issues associated with obtaining written informed consent. This reduced the number of patients treated on an emergency basis and may also have mitigated the role of contrast-induced postoperative AKI.

## Conclusions

In this pooled analysis of 2 randomized clinical trials, glutamate infusion was associated with a significantly lower risk of AKI after CABG among patients without diabetes. The results are exploratory and need to be confirmed in adequately powered prospective randomized trials. Such efforts are important given the prognostic implications of AKI and the current lack of evidence-based treatment.

## Notes

Supplement 1.
Trial Protocols

Supplement 2.
Data Sharing Statement
