# Error in Table 2

**DOI:** 10.1001/jamanetworkopen.2026.1997

**Published:** 2026-02-20

In the Original Investigation titled “Association of Glutamate Infusion With Risk of Acute Kidney Injury After Coronary Artery Bypass Surgery: A Pooled Analysis of 2 Randomized Clinical Trials,”^1^ published January 22, 2024, the Kidney Disease Improving Global Outcomes (KDIGO) data presented in Table 2 are inaccurate because the sampling protocol did not comply with the time windows of KDIGO; therefore, the table row providing data on KDIGO stage 1 or higher has been removed. This article has been corrected.^1^
