# Supplementary material for: Association of Glutamate Infusion With Risk of Acute Kidney Injury After Coronary Artery Bypass Surgery: A Pooled Analysis of 2 Randomized Clinical Trials
Source: JAMA Netw Open. 2024 Jan 22;7(1):e2351743. doi: 10.1001/jamanetworkopen.2023.51743 (PMC10804267; doi:10.1001/jamanetworkopen.2023.51743)
Supplement: Supplement 1. — Trial Protocols [file jamanetwopen-e2351743-s001.pdf]

GLUTAMICS I ORIGINAL STUDY PROTOCOL AND APPLICATION TO THE SWEDISH MEDICAL PRODUCT AGENCY + AMENDMENTS – TRANSLATED FROM SWEDISH LANGUAGE

Metabolic protection of the heart with glutamate in association with surgery for unstable coronary artery disease (GLUTA*MICS* – GLUTAmate for Metabolic Intervention in Coronary Surgery)

Principal Investigator: Rolf Svedjeholm (MD, PhD, Associate Professor) Dept of Cardiothoracic and Vascular Surgery, University Hospital, SE581 85 Linköping

Telephone: 013-224825
E-mail: rolf.svedjeholm@lio.se

Site: Dept of Cardiothoracic and Vascular Surgery, University Hospital, SE581 85 Linköping

Background

Great efforts have been made to protect the heart during cardiac surgery. In particular, they have focused on the period when the heart is disconnected from the circulation. With modern myocardial protection, the heart can now be cut off from circulation for several hours. Despite the relatively short cross-clamp times in coronary surgery, almost 10% of patients suffer a myocardial infarct or significant myocardial damage. The reason for this is that preoperative and to some extent postoperative ischemia remain important risk factors for the development of myocardial injury. One group particularly exposed to the risk of preoperative ischemia is patients undergoing surgery for unstable angina pectoris.

In addition to traditional pharmacological anti-ischemic treatment, there is the possibility of treating these patients metabolically. It is possible to use the heart's ability to adapt metabolically to ischemia. Coronary artery disease patients have an increased uptake of the amino acid glutamate. This amino acid plays a key role in the metabolism of heart muscle cells. In animal experiments, it has been found that glutamate increases the heart's ischemia tolerance and stimulates cardiac recovery after ischemia. Biochemically, this has been explained by the fact that glutamate can contribute to the generation of anaerobic energy in the citric acid cycle via the metabolism of alpha-ketoglutarate to succinate, and that anaerobic glycolysis in the cytosol is stimulated via the malate-aspartate bundle, which contributes to the preservation of the redox potential in the cell's cytosol. Lactate accumulation is counteracted by transamination of pyruvate to alanine. After ischemia, glutamate helps to restore depleted citric acid cycle metabolites and may thus accelerate the normalization of oxidative metabolism which may be disturbed after severe ischemia.

In human studies, cardiac metabolism in patients with angina pectoris has been found to be characterized by an increased uptake of glutamate and an increased release of alanine. In patients

with angina pectoris, glutamate supplementation has been shown to delay the onset of chest pain and ST depression during exercise testing and pacing.

In a randomized double-blind study in patients undergoing surgery for unstable angina necessitating infusion of nitroglycerine, we found metabolic evidence of better ischemia tolerance before surgery, and earlier normalization of cardiac metabolism after surgery, in patients receiving pre- and peroperative glutamate infusion.

Objective

The objective of this clinical trial is to study whether the above characteristics allow glutamate infusion to limit the ischemic myocardial damage and thus reduce the risk of death and heart failure in association with surgery for unstable angina.

Timetable

In the fall of 2005, recruitment of patients is expected to begin in Linköping, Örebro and Karlskrona. Possibly Umeå will join at a somewhat later stage. The study is expected to be completed in about 2-3 years.

Type of trial

Externally randomized, placebo-controlled study with parallel study groups and double-blind masking. Placebo will be a physiological saline solution which, like the glutamate solution, is a clear solution.

Study population

Patients accepted for coronary surgery due to unstable coronary artery disease.

The above patients will be informed and asked to participate in the study. Patients who accept participation will be included in the study. Patients who require emergency surgery (cannot wait 24 hours) will be analyzed separately.

Exclusion criteria: Patients with unclear food allergy or asthma, patients weighing > 120 kg, patients who are in such a poor condition that they cannot be asked to participate or patients who require inotropic drugs or mechanical circulatory support (aortic balloon pump) already before being included in the study.

Linköping, Örebro and Karlskrona perform all coronary artery surgery within their respective catchment areas and the above inclusion criteria capture about 90% of the patients who undergo coronary artery surgery for unstable coronary artery disease. To evaluate the consequences of non-response, the population that is not included in the study can be analyzed via the clinic's data register where it should be stated whether the patient participated in the study or not

Research plan

Randomized double-blind study in which 2214 patients undergoing surgery for unstable angina will be included. Half of the patients will receive a glutamate infusion (1.65 ml/kg per hour of 0.125 M L-glutamate solution) which will be started at the beginning of anesthesia and surgery. The infusion is stopped when the heart-lung machine is started and resumed when reperfusion is started (removal of the aorta forceps) and is then given for 2 hours. Then another 50 ml of the solution is given with the infusion rate reduced to half. A maximum of 500 ml of solution is given. Other patients receive an infusion of physiological saline according to a similar schedule.

Patients are assessed for mortality, myocardial injury markers, clinical course and need for circulatory support.

Sample size

The sample size is based on available results from our department of cardiothoracic surgery for patients operated on for unstable angina 1995-2000 (mortality 2.8%; perioperative myocardial infarction 6.9%: postoperative heart failure 7%; all 12.3%). The power calculations have been made in consultation with statistical experts (80% power; 5% risk level; two-sided test; 30% reduction of an event occurring in 12% giving 1107 patients/group) and assume that metabolic prophylaxis with glutamate could reduce complications by 30% and that the expected incidence of the composite endpoint is 12%. Interim analysis will be performed after one year (800 patients) to assess whether there is support for continuation. (NOTE – the trial was terminated after interim analysis)

Primary endpoint:

a) Demonstrate differences in the composite endpoint consisting of mortality (30-day + hospital mortality) + perioperative myocardial infarction + postoperative heart failure.

Secondary endpoints:

a) Demonstrate differences in the extent of ischemic myocardial injury (CK-MB, troponin-T, ECG) (see criteria in research program).

b) Demonstrate differences in the need for circulatory support (use and dose of inotropic drugs and mechanical circulatory support).

c) Demonstrate differences in postoperative circulatory status (invasive arterial pressure, central ventricular pressure, pulmonary arterial pressure, mixed venous-central venous oxygen saturation).

d) Demonstrate any differences in the need for intensive care, length of stay, ventilator care, incidence of atrial fibrillation, secondary complications on vital organs (kidneys, lungs, liver, CNS).

Substudies:

a) Patients with markedly impaired left ventricular function (EF< 0.35) will be studied in terms of hemodynamics preoperatively and postoperatively (Swan-Ganz, Echocardiography) b) The effect of intravenous glutamate infusion on renal function measured by p-Cystatin C in patients operated for unstable angina pectoris c) The effect of intravenous glutamate infusion on S100 B in patients operated for unstable angina pectoris. Subgroup analyses will be performed in patients with severe left ventricular dysfunction, diabetics, women, CABG + valve and patients requiring intravenous infusion of nitroglycerine and urgent surgery (< 24 hours).

Patient information and informed consent

All patients who meet the inclusion criteria for the study receive oral and written information about the study. Written consent is obtained in which the patient also agrees to any future quality control by independent reviewers.

Ethical considerations

Myocardial infarction is one of the most important complications of coronary artery surgery. The risk is particularly high if the patient is operated on because of impending ischemia (lack of oxygen) in the heart. Myocardial infarction is the leading cause of heart failure after surgery and the leading cause of death in this group. There is currently evidence that glutamate increases the heart's tolerance to ischemia (lack of oxygen) and that the risk of myocardial infarction and the size of the infarct can be limited by glutamate. The potential benefits of the study are therefore considered to be great.

The patients will receive normal care in addition to the infusion of 300 - 500 ml of glutamate solution or saline solution.

Glutamate is a naturally occurring amino acid and the most abundant free amino acid in heart muscle cells. In ischemia, glutamate is depleted, and the heart attempts to replace it by taking up glutamate from the blood. The extraction rate is about 50%. Glutamate levels rise rapidly with both oral and intravenous administration. In isolated individuals, transient discomfort (Chinese restaurant syndrome: flushing, face-head pain, chest pressure) has been reported. However, despite very extensive use of this substance, there is no evidence that exogenous administration has caused nerve damage or other permanent complications in humans or primates. One reason for this may be that glutamate does not cross the blood-brain barrier. It should also be noted that glutamate levels in the central nervous system have been reported to be up to 50 times higher than in blood. The glutamate infusion given to patients increases whole blood levels 2-3 times, which has been shown to double the uptake of glutamate by the heart. Higher infusion rates do not appear to provide a further increase in glutamate uptake.

Clinical significance and clinical experience

Our patients may benefit from the study in terms of reduced mortality and less risk of serious complications such as myocardial infarction and postoperative heart failure. According to O'Connor, postoperative heart failure was the primary cause of 2/3 of deaths after cardiac surgery in Northern New England. Our group has utilized glutamate in clinical practice for more than ten years and the experience so far shows a mortality rate that has been 50% lower than expected in coronary surgery. Compared with national and international results, we have noted that the risk of kidney failure (considered the most sensitive indicator of the quality of heart failure treatment) after coronary surgery has been at least 5 times lower (regardless of the outcome variable considered).

Work with different risk assessment protocols suggests that surgical quality is most easily assessed in low-risk groups, while it is likely that the quality of perioperative care has a greater impact on outcomes in high-risk groups. Patients with preoperatively severely impaired left ventricular function is the group where we previously used glutamate as a preventive measure to the greatest extent. In a yet unpublished study involving more than 100 patients, we recorded a mortality of 1.0% in a cohort where the expected risk-corrected mortality according to logistic Euroscore was 8%. As these types of results are affected by other factors, they must be interpreted with caution. Our power calculations for the planned study are therefore based on a more conservative estimate where we have calculated a 30% reduction in the primary endpoint, which is a composite of mortality (30 days + hospital), perioperative myocardial infarction and postoperative heart failure.

The biochemical properties of glutamate imply that beneficial effects demonstrated in this study should be reproducible in studies involving other groups of patients with acute ischemic heart disease. The main future clinical significance may therefore prove to be in the cardiological treatment of acute myocardial infarction and other conditions with acute coronary syndromes.

Route of administration of glutamate

Glutamate levels rise rapidly with both oral and intravenous administration. In cardiac surgery, glutamate has been administered via cardioplegia solution (additive) or intravenously. The reasons for the choice of intravenous administration in the current study are: 1) to address the preoperative ischemia 2) There are experimental data suggesting that the transport of glutamate across the cell membrane is inhibited by cardioplegia (hyperkalemic solution) and that the heart muscle cells instead leak glutamate in the cardioplegic state.

Dosage of glutamate

Available data suggest that cardiac uptake of glutamate is strongly correlated with arterial levels in patients undergoing coronary artery surgery. It is also known that cardiac uptake of glutamate increases when plasma levels are raised by intravenous administration. However, an increase in arterial levels of more than 2-3 times has not been found to provide a further increase in cardiac glutamate uptake. These arterial levels are achieved by infusion of 1.65 ml/hour per kg body weight of a 0.125 M glutamic acid solution.

The total infusion volume according to the planned infusion schedule (see research plan) is as follows depending on the weight of the patient: 60 kg - 314 ml; 70 kg - 358 ml; 80 kg - 402 ml; 90 kg 457 ml; 100 kg - 500 ml. The calculations assume that it takes about 45 minutes from the start of the infusion until the heart-lung machine can be started. The maximum amount infused is 500 ml. Patients weighing more than 100 kg will receive a slightly shorter infusion during the reperfusion phase and to avoid major discrepancies, patients weighing more than 120 kg will not be included in the study.

Details on the composition of the solution can be found in the pharmaceutical documentation.

Handling of medicines

Drug management is done according to the recommendations of the manufacturer Apoteksbolaget.

Randomization, Control treatment, Patient identification

Randomization is conducted externally at Apoteksbolaget in Umeå where the preparation of the solutions also takes place.

The masking is double-blind. Placebo in the study consists of physiological saline solution. Both the glutamate solution and the placebo are clear transparent solutions that are indistinguishable to the eye. The bottles are labeled with a patient code that is registered in the CRF and the clinic's data register. A patient identification list will be maintained to allow identification of patients for data quality control purposes.

The randomization code will be stored at Apoteksbolaget in Umeå. It should be possible to break the code for an individual patient in case of suspected adverse effects. This is done by the person who is primarily responsible for the preparation of solutions/randomization at the request of the investigator, or a person delegated by the investigator.

Adverse reaction reporting

Suspected adverse events will be reported to the Swedish Medicines Agency in accordance with the regulations issued by the Agency. Suspected side effects or other adverse events during the study will also be reported to the local ethics committee.

Monitoring, quality control and interim analyses

Internally, the study will be monitored by weekly responsible employees to continuously ensure adequate inclusion procedure, complete sampling, archiving and checking registered CRFs and to register dropouts.

Monitoring for adverse events will be done continuously by an external research nurse. Monitoring for quality control of data will be done intermittently by an external monitor.

External statistical expertise from the Department of Mathematics at Linköping University will perform interim analysis, which is planned to take place after 800 patients have been included (see below).

The Swedish Medical Products Agency will be given the opportunity to perform/order independent external monitoring.

Reasons for premature termination of the study

The study will be continuously monitored by an external research nurse. If this monitoring or interim analysis provides evidence of a suspected increased risk of stroke or a negative effect on mortality, the study will be terminated early. If continuous monitoring should provide evidence of unexpected and serious adverse events in addition to what is stated above, the study will also be discontinued. The safety aspects will thus be prioritized and the trial should be able to be terminated prematurely before the occurrence of any side effects becomes statistically significant. A positive treatment effect on mortality at interim analysis will also be a stopping criterion, but this requires statistical significance ($p<0.05$). The study will also be stopped for resource reasons if there is no absolute difference in favor of the treatment group regarding the primary end-point.(NOTE – the study was prematurely terminated after interim analysis because of futility regarding the primaary endpoint)

Insurance

Cardiac surgery carries risks of complications, pain and suffering. It is very unlikely that the study would adversely affect this. Patients' insurance coverage is therefore provided by the existing patient insurance.

Information of staff

All relevant categories of staff will be informed orally about the study, its background and purpose. Contact persons will be appointed who will continuously answer questions that arise and, in addition, written information about the study will be readily available at all relevant healthcare units.

Sponsor

Rolf Svedjeholm, Thoraxkir sektionen, Thorax-kärlkliniken, US, Linköping

Investigators

Rolf Svedjeholm, Thoraxkir sektionen, Thorax-kärlkliniken, US, Linköping

Erik Håkansson, Thoraxanestesi, Thorax-kärlkliniken, US, Linköping
Jonas Holm, Thoraxkir sektionen, Thorax-kärlkliniken, US, Linköping

Örjan Friberg, Thoraxkir sektionen, Thoraxkliniken, US, Örebro
Lena Sunnermalm, Thoraxanestesi, Thoraxkliniken, US, Örebro
Mårten Vidlund, Thoraxkir sektionen, Thoraxkliniken, US, Örebro

Sören Juhl-Andersen, Thoraxanestesi, Thoraxkliniken, Blekingesjukhuset, Karlskrona
Jan Olov Borg, Thoraxkir sektionen, Thoraxkliniken, Blekingesjukhuset, Karlskrona

Distribution of responsibilities and applicable delegations within the trial team

The principal investigator is responsible for delegating tasks within the trial to different staff members. Considering the length of the study period and that employees can be added / leave, delegations will be continuous and limited in time.

Reporting and publication

The results of the study will be made available regardless of outcome and submitted for peer-review to internationally leading journals in the field of circulation / cardiology / cardiac surgery. The choice of journal will be discussed within the trial team when the results are ready. Pre-presentation of the results may be relevant at an international congress in the field of circulation.

Archiving of trial material

Primary data will be archived and available for independent external monitoring for 10 years. Archiving will be done in a way that does not compromise patient confidentiality and integrity. CRFs will be kept locked and CRFs should not allow identification of individual patients other than with access to the patient code and identification list kept in separate locked areas.

References

1. Svensson SE, Svedjeholm R, Ekroth R, Milocco I, Nilsson F, Sabel K G, William-Olsson G. Trauma metabolism and the heart; uptake of substrates and effects of insulin early after cardiac surgery. J Thorac Cardiovasc Surg 1990; 99: 1063 - 1073.
2. Svedjeholm R, Svensson SE, Ekroth R, Milocco I, Nilsson F, Vinnars E, Wernerman J. Trauma metabolism and the heart; studies of heart and leg amino acid flux after cardiac surgery. Thorac Cardiovasc Surgeon. 1990; 38: 1-5.
3. Svedjeholm R, Ekroth R, Joachimsson PO, Ron-quist G, Svensson SE, Tydén H. Myocardial uptake of amino acids and other substrates in relation to myocardial oxygen consumption four hours after cardiac surgery. J Thorac Cardiovasc Surg 1991; 101: 688 - 694.
4. Svedjeholm R, Ekroth R, Joachimsson PO, Tydén H. High dose insulin improves the efficacy of dopamine early after cardiac surgery. A study of myocardial performance and oxygen consumption. Scand J Thorac Cardiovasc Surg. 1991; 25: 215 - 221.
5. Svedjeholm R, Hallhagen S, Ekroth R, Joachimsson PO, Ronquist G. Dopamine and High-dose Insulin infusion (Glucose-Insulin-Potassium) after a Cardiac Operation. Effects on Myocardial Metabolism. Ann Thorac Surg 1991; 51: 262 - 270.
6. Svedjeholm R, Huljebrant I, Håkanson E, Vanhanen I. The Rationale for Metabolic Support with Amino Acids and GIK (Glucose-Insulin-Potassium) in Cardiac Surgery. Ann Thorac Surg.1995; 59:S15-22.
7. Svedjeholm R, Huljebrant I, Håkanson E, Vanhanen I. Glutamate and high-dose glucose-insulin-potassium (GIK) in the treatment of severe cardiac failure after cardiac operations. Ann Thorac Surg. 1995; 59: S23-30.
8. Svedjeholm R, Vanhanen I, Håkanson E, Joachimsson PO, Jorfeldt L, Nilsson L. Metabolic and hemodynamic effects of intravenous glutamate infusion early after coronary surgery. J.Thorac.Cardiovasc.Surg. 112(6):1468-1477, 1996.
9. Vanhanen I, Svedjeholm R, Håkanson E, Joachimsson PO, Jorfeldt L, Nilsson L, Vanky F. Assessment of myocardial glutamate requirements early after coronary artery bypass surgery. Scand Cardiovasc J 1998; 32: 145-152.
10. Vanhanen I, Håkanson E, Jorfeldt L, Svedjeholm R. Myocardial uptake and release of substrates in patients operated for unstable angina: impact of glutamate infusion. Scand Cardiovasc J 2003; 37: 113-120.

AMENDMENT 1

Linköping 2005-10-10

Docent Göran Sundlöf
Läkemedelsverket
Kliniska prövningar – Licenser
PO Box 26
751 03 Uppsala

(Swedish Medical Product Agency)

Supplement to the trial protocol for Clinical trial (Dnr: 151:2003/70403) "Metabolic protection of the heart with glutamate during surgery for unstable angina pectoris".

The start of the above-mentioned study has been delayed due to an ongoing study at the cardiology department on the same patient group. During the waiting period, two additional centers have been involved and some minor changes have been made to the trial protocol. The transformation of the study into a multicenter trial and the current protocol changes have been approved by the Ethical Review Board (copy of EPN decision Dnr: M87-05 is attached). Resource certificates from the respective principals have also been obtained. A new trial protocol and investigator's manual where code-breaking routines and endpoints are specified in more detail is attached. The most important changes are summarized and justified below:

The title of the study has been modified: the term unstable angina has been replaced by the term unstable coronary artery disease, which gives a more accurate description of the study population. The study has also been given an English abbreviation GLUTAMICS, which stands for GLUTAmate for Metabolic Intervention in Coronary Surgery.

The study has been transformed into a multicenter study involving Linköping, Örebro and Karlskrona. Details of this and investigators are now in the trial protocol. Possibly Umeå will also join. In this case, the name of the investigators will be communicated to the Swedish Medical Product Agency.

The exclusion criteria have been modified. Age over 80 years is no longer a reason for exclusion. The reason for this is that patients over 80 years of age have become so common that the risk of skewing the material is now considered small.
Patients operated on without a heart-lung machine are now included in the primary analysis but will also be analyzed separately. Other planned subgroup analyses have been further specified. These changes are approved by the EPN (Ethical Review Board). Adverse event reporting has been further clarified in the protocol and a copy of the FAX form for reporting to the sponsor is attached.

Sincerely

Rolf Svedjeholm
Överläkare, docent
Thorax-kärlkliniken
Universitetssjukhuset
581 85 Linköping

tel: 013-224825

rolf.svedjeholm@lio.se

AMENDMENT 2

Linköping 2007-03-19

Docent Göran Sundlöf
Läkemedelsverket
Kliniska prövningar – Licenser
PO Box 26
751 03 Uppsala
(Swedish Medical Product Agency)

Addendum to the trial protocol for Clinical Trial (Dnr: 151:2003/70403) "Metabolic protection of the heart with glutamate in surgery for unstable angina pectoris" and resulting corrections in the Investigator's Manual for GLUTAMICS.

In October 2005, recruitment of patients began in Linköping. Örebro and Karlskrona have included patients since November 2005. Other consulted clinics have declined to participate for various reasons. The proportion of patients with unstable coronary artery disease who go to surgery has decreased in recent years in line with the development of PCI. However, this trend now seems to have been broken and a tendency in the opposite direction has been noted, probably due to recent alarm reports on DES stents. Today, almost 400 patients have been included. GLUTAMICS is thus already one of the largest investigator-initiated clinical trials in cardiac surgery. Despite this, the fact remains that the main study may take another 5-6 years to complete assuming last year's inclusion rate. **At investigator meetings, the implementation of the study has therefore been discussed and subsequently clarified in the trial protocol:**

GLUTAMICS will be a PhD project and results will be reported from both the main study and substudies. Results regarding surrogate end-points myocardial injury markers, heart failure markers, brain injury markers and kidney function markers will be reported after interim analysis, which is expected to take place in just over a year.

Research funds allocated so far can thus also be used in a more efficient way, which among other things allows room for continued external professional monitoring according to GCP standards.

With a more reliable power calculation after the interim analysis regarding the primary end-point and a positive outcome regarding the above-mentioned surrogate end-points, the investigator group believes that the project will be able to generate increased research funding and interest to motivate more cardiac surgery clinics to participate in GLUTAMICS.

New investigator: Dr. Farkas Vanky has been included as a new investigator in Linköping and this has been added to the protocol.

The exclusion criteria have been modified:
Age over 85 years has been added as an exclusion criterion since these patients are very few and since the clinical course of these patients is often long and complicated even if the heart copes well with the operation.

Preoperative dialysis has been added as an exclusion criterion. The reason for this is that preoperative dialysis is rare and that dialysis itself is an endpoint in the study.
The upper weight limit of the study has been adjusted upwards from 120 kg to 125 kg. The reason for this is that we found that there are quite a few patients who weigh just over 120 kg.

The investigator's manual has also been updated due to the above adjustments of the exclusion criteria. The inclusion criteria in the investigator's manual have been clarified so that it is clear that all patients with unstable coronary artery disease that can be classified according to Braunwald can be included regardless of whether the surgery is done with or without a heart-lung machine, or if the procedure also includes valve surgery.

Clarification on AE and SAE reporting in the examiner's manual
The guidelines for AE / SAE reporting have been clarified in the Investigator's Manual. Considering that the study is conducted on patients undergoing major cardiac surgery, it can be expected that a significant proportion of the patients are affected by some form of adverse event and that it is often a question of SAE. According to a previous agreement between the sponsor and Göran Sundlöf at the Medical Product Agency, the sponsor should not report each individual SAE to the Medical Product Agency if it is not a SUSAR. The investigator registers AE and SAE and reports mortality, stroke < 24 hours from surgery + SUSAR to the sponsor. Sponsor reports SUSAR to the Medical Product Agency. In case of mortality or stroke < 24 hours from surgery, the sponsor is obliged to ensure that the code is opened and registration takes place.

However, before code breaking, the investigator should have decisions on all primary endpoints in the CRF. If there is a deviation between the groups such that the stopping criteria in the protocol are met, the sponsor should inform the Medical Product Agency.

There is currently 1 case of stroke registered in the treatment group and 4 cases of stroke in the placebo group. Regarding mortality, there are currently 2 known cases in both groups.

The criteria for a variable included in the primary endpoint, postoperative left ventricular failure at weaning from cardiopulmonary bypass, have been modified. The problem is that there is no generally accepted definition. The investigator's manual was originally written with an attempt to find clear, subsequently verifiable criteria (relationship between venous oxygen saturation and systemic blood pressure or cardiac index / systemic blood pressure) in conjunction with clinical assessment by the responsible anesthesiologist. With this wording in the examiner's manual, it was mandatory that the responsible anesthesiologist had assessed that failure was present for the condition to be classified as postoperative heart failure. However, it has turned

out that several cases of obvious heart failure were misclassified as "non-failure" and vice versa, probably due to human error / time pressure when entering data in Carath (the clinics' common database).

It is also evident that it may be easier to determine in retrospect whether postoperative heart failure has occurred when one has access to the entire course of events compared to the early postoperative situation when data are recorded in Carath under time pressure. To overcome this problem, it was decided at a clinical investigator meeting in Örebro on October 24, 2006 that the clinical assessment should be done by consensus discussion within the investigator group. In this assessment, a blinded assessment of all relevant data available in the medical record and the Carath database are considered (anesthesiologist's assessment, surgeon's assessment, hemodynamic data, information on echocardiographic findings, clinical course and need for circulatory support). All cases where heart failure cannot be clearly excluded should be taken to a consensus round. Consequently, the heart failure criteria have been corrected in the investigator's manual: For the patient to be classified as heart failure in the CR, a clinical assessment by the consensus group + the fulfillment of one of the criteria b, c or d for postoperative left ventricular failure at weaning from CPB t is required.

In the first two consensus rounds, 28 cases were settled, with full consensus reached in all but two cases, which were settled by vote.

At the second consensus round in Linköping on January 30, 2007, it became apparent that there were several cases of postoperative cardiac and circulatory failure with late onset.
During the discussion, it emerged that this needs to be clarified and that we therefore need to introduce another option as a secondary endpoint, namely **late circulatory failure**.

By definition, these are patients who have had uncomplicated machine departure without signs of failure but subsequently developed circulatory impairment for any reason in the intensive care unit / ward (bleeding, tamponade, arrhythmias, sepsis or for unclear reasons) with or without secondary impact on cardiac function. Late circulatory failure will be analyzed separately as a secondary endpoint and to facilitate future work, the cases are divided into the options **Cardiac cause** where the cause is obviously primarily cardiac and **Other causes** which also includes cases with uncertain but non-cardiac genesis.

The consensus round also decides whether the criteria for the secondary end-point "severe failure" are met if the patient is classified as "postoperative failure at weaning from CPB" or "late circulatory failure". In connection with the consensus round, a decision must also be made as to whether mortality was cardiac or of other cause.

Against this background, we also intend to **add a substudy to the protocol**. The reason for the study addition (substudy d in the trial protocol) is that there are currently no accepted criteria for postoperative heart failure. However, it has been shown that elevation of natriuretic peptides correlates well with the degree of heart failure and prognosis in non-surgical patients. Data from cardiac surgery are limited but point in the same direction.

The objective of this study extension is to investigate if there is a correlation between **NT-proBNP** and the heart failure criteria applied in the study and to investigate if glutamate infusion affects the postoperative levels of NT-proBNP. Sampling is planned on the remaining 400 patients for interim analysis. This represents twice as many patients as any previous cardiac surgery study on natriuretic peptides has included so far and should, according to our local expert in the field (Dr. Urban Alehagen), be sufficient to demonstrate associations between markers and clinical heart failure criteria and indicate any clinically relevant effects of the intervention. The application for approval of this study extension has also been sent to EPN (Ethical Review Board) in Linköping.

To make changes easy to find, copies of the study protocol and investigator's manual are also attached with the changes highlighted in yellow.

Sincerely.

Rolf Svedjeholm
Överläkare, docent
Thorax-kärlkliniken
Universitetssjukhuset
581 85 Linköping
tel: 013-224825

rolf.svedjeholm@lio.se

AMENDMENT 3

Linköping 2007-09-17

Docent Göran Sundlöf
Läkemedelsverket
Kliniska prövningar – Licenser
PO Box 26
751 03 Uppsala
(Swedish Medical Product Agency)

Addendum to the trial protocol for Clinical Trial (Dnr: 151:2003/70403) "Metabolic protection of the heart with glutamate in surgery for unstable angina pectoris" and resulting corrections in the Investigator's Manual for GLUTAMICS.

At the investigator meeting 2007-08-23 in Karlskrona, the following amendments to the above study were decided.

New investigator / change of principal investigator Karlskrona: The study has suffered a major loss. The principal investigator in Karlskrona Dr Sören Juhl Andersen has passed away and this causes the following changes:

Dr Rajiv Sharma, Chief Physician in Anesthesia, Thoracic Clinic, Blekinge Hospital in Karlskrona is the new investigator in the study as of 2007-08-23. Dr Jan-Olov Borg takes over as principal investigator in Karlskrona.

The exclusion criteria have been modified:

Addition of exclusion criteria.

Unforeseen significant expansion of the surgical procedure that greatly overshadows the dignity of the planned surgery (e.g. aortic ascending surgery, severe bleeding complications intraoperatively)

Background: Some patients included in the study have had the study drug stopped by the physician responsible for the patient due to unforeseen severe expansion of the procedure (aortic dissection in connection with cannulation for heart-lung machine, aortic ascending surgery, severe bleeding problems from fragile ruptured aorta). This was discussed at the investigators' meeting, and it was concluded that this should be set up as a formal exclusion criterion in the study, i.e.: that the study infusion can be closed and the patient excluded in the event of unforeseen significant extension of the procedure that overshadows the planned operation (eg aortic ascending surgery, severe bleeding complications intraoperatively)

Modification of secondary endpoint "Severe postoperative heart failure" in the investigator's manual:

"Severe postoperative heart failure" should also include patients who had severe heart failure and died even if they had not been treated >for 48 hours in intensive care (in the previous writing, this obvious relationship was neglected).

Sincerely,

Rolf Svedjeholm
Sponsor för GLUTAMICS
Överläkare, docent
Thorax-kärlkliniken
Universitetssjukhuset
581 85 Linköping
tel: 013-224825

rolf.svedjeholm@lio.se

GLUTAMICS II ORIGINAL STUDY PROTOCOL AND APPLICATION TO THE SWEDISH MEDICAL PRODUCT AGENCY + AMENDMENTS + STATISTICAL ANALYSIS PLAN – TRANSLATED FROM SWEDISH LANGUAGE

Metabolic intervention with glutamate in surgery for ischemic heart disease:(GLUTA*MICS* II = GLUTAmate for Metabolic Intervention in Coronary Surgery II)

EudraCT nummer: 2011-006241-15

Sponsor's Protocol Code Number GLUTAMICS2

Sponsor och Principal Investigator: Rolf Svedjeholm (överläkare, professor) Thorax-kärlkliniken, Universitetssjukhuset, 581 85 Linköping

Telefon: 010-1034825
E-post: rolf.svedjeholm@lio.se

Study sites:

Thorax-kärlkliniken, Universitetssjukhuset, Linköping

Thoraxkliniken, Universitetssjukhuset, Örebro

Kliniken för thoraxkirurgi, anestesi och intensivvård, Skånes Universitetssjukhus, Lund.

There is preliminary interest from other Swedish cardiac surgery clinics if sufficient funding can be obtained. Please see timetable.

Synopsis

GLUTAMICS II (EudraCT number: 2011-006241-15) is an externally randomized, placebo-controlled trial with parallel study groups and double-blind masking. The study drug consists of 0.125 M glutamate solution given intravenously in connection with coronary artery surgery. Placebo consists of physiological saline. The aim is to confirm findings made in subgroups in a previous study GLUTAMICS and show that intravenous glutamate infusion in coronary artery surgery reduces the risk of serious postoperative heart failure.
The primary endpoint is a composite of a) cardiac mortality (30-days + hospital mortality) b) significant postoperative heart failure according to pre-specified criteria c) postoperative renal failure (new dialysis requirement or duplication of p-creatinine). The study population consists of patients with CCS class IV angina or moderate-severe left ventricular function undergoing coronary artery surgery with a heart-lung machine.

Exclusion Criteria: Patients with unclear food allergy such as triggered shortness of breath, urticaria or flush; patients with diabetes, patients > 85 years of age, patients who have had previous heart surgery, patients who are in such poor condition that they cannot be asked to participate, patients who for linguistic or other reasons cannot give informed consent, patients on preoperative dialysis or p-creatinine >250 µmol/l, patients requiring inotropic drugs or mechanical circulatory support (intra-aortic balloon pump) due to circulatory failure even before being included in the stud, patients undergoing concomitant valve surgery or fibrillation ablation. Unforeseen significant expansion of the surgical intervention that greatly overshadows the dignity of the planned operation.
1400 patients will be included preliminarily, and interim analysis is planned after 800 patients. The study is expected to start in 2013 and be completed after 2-3 years. (NOTE: major modifications of the trial in Amendment 1)

Background

Postoperative heart failure remains the leading cause of prolonged intensive care, multi-organ failure and death after heart surgery. Conventional treatment of postoperative heart failure with inotropic drugs improves hemodynamics, but at the cost of an excessive increase in myocardial oxygen consumption, which can aggravate the underlying heart muscle damage.

Cardiac metabolism in coronary artery disease patients is characterized by an increased uptake of the amino acid glutamate. This amino acid plays a key role in the metabolism of heart muscle cells. Animal experiments, glutamate has been found to increase tolerance to myocardial ischemia.* After ischemia, glutamate plays an important role in restoring citric acid cycle metabolites consumed in the mitochondria and can thus stimulate the normalization of oxidative metabolism that is disturbed after severe ischemia.

** Biochemically, this is explained by the fact that glutamate can contribute to the generation of anaerobic energy in the citric acid cycle via the metabolism of alpha ketoglutarate to succinate and that the anaerobic glycolysis in the cytosol is stimulated via the malate - aspartate hunt that helps to preserve the redox potential in the cytosol of the cell. Lactate accumulation is counteracted by transamination of pyruvate to alanine.*

Even in smaller human studies, a positive effect of glutamate on the metabolic and functional recovery of the heart has been found. Based on this and many years of encouraging clinical experiences where we achieved results in high-risk patients unsurpassed in the literature both in terms of survival and heart failure-related complications, the first GLUTAMICS study was initiated.

In this multicenter study, 861 patients undergoing coronary artery surgery due to acute coronary syndrome were randomized to blinded intravenous infusion of glutamate or placebo (saline). The primary endpoint was a composite of postoperative mortality (≤ 30 days), perioperative myocardial infarction and left ventricular failure during weaning from cardiopulmonary bypass. The results showed no difference in primary endpoint between the groups.

Regarding secondary endpoints, there were significant differences suggesting a beneficial effect of glutamate on cardiac recovery after ischaemia. There were significantly fewer patients in the glutamate group who developed severe heart failure that prolonged the intensive care period or required treatment with intra-aortic balloon pump. The presumed efficacy of glutamate infusion was seen in patients with the most severe angina (CCS class IV) who constituted a prespecified subgroup. A relative risk reduction in excess of 50% was also seen in other high-risk groups (see Table 1) with the exception of diabetics. Diabetes has subsequently been shown to be associated with a downregulation of glutamate transporters in the mitochondria and greatly elevated intracellular glutamate levels, which could explain the lack of effect of glutamate administration.

The advantage of glutamate is that it works by stimulating the natural recovery mechanisms, which could be less stressful than conventional treatment with inotropic drugs that increase myocardial oxygen consumption and can aggravate the underlying heart muscle damage. In patients who had signs of heart failure at weaning from cardiopulmonary bypass, we accordingly found a significantly shorter intensive care time and fewer patients with renal failure in the glutamate group (Table 2).

The first GLUTAMICS study was initially planned for patients with unstable angina, but the inclusion criteria were later broadened to include all patients with acute coronary syndrome, which led to the inclusion of many low-risk patients which diluted the results and reduced the study's power. We also found that the primary endpoint had benefited from a more robust design regarding the postoperative heart failure criteria, which were clouded by liberal use of preemptive inotropic treatment.

The results thus suggest that intravenous glutamate infusion could be an important and prudent way to promote post-ischemic recovery of the heart, which might also become important in cardiology practice. For intravenous glutamate infusion to initially become widely available and approved for the treatment and prophylaxis of heart failure after coronary artery surgery, the results must be confirmed in a new trial.

Objective

The aim is to confirm in a clinical trial that glutamate infusion in connection with coronary artery surgery reduces the risk of developing postoperative heart failure leading to death, kidney failure or prolonged intensive care.

Type of trial

Externally randomized, placebo-controlled study with parallel study groups and double-blind masking. Placebo will consist of physiological saline which, like the glutamate solution, is a clear transparent solution.

Study population

Patients accepted for coronary artery surgery who are considered to be at increased risk of postoperative heart failure:

- unstable angina (CCS class IV)
- moderate – pronounced impaired left ventricular function preoperatively (Ejection fraction ≤ 0.45)

The above patients will be informed and asked about participation in the study. Patients who accept participation and are operated on with a heart-lung machine are included in the study.

Exclusion Criteria: Patients with unclear food allergy which has triggered shortness of breath, urticaria or flush; patients with diabetes, patients > 85 years of age, patients who have had previous heart surgery, patients who are in such poor condition that they cannot be asked to participate, patients who for linguistic or other reasons are unable to give informed consent, Patients on preoperative dialysis or p-creatinine >250 µmol/l, patients requiring inotropic drugs or mechanical circulatory support (intra-aortic balloon pump) due to circulatory failure prior to their inclusion in the study, patients undergoing concomitant valve surgery or fibrillation ablation. Unforeseen significant expansion of the surgical procedure that greatly overshadows the dignity of the planned operation (e.g. aortic ascending surgery, severe bleeding complications intraoperatively). (NOTE - exclusion criteria modified in the final protocol)

Linköping and Örebro perform all coronary artery operations in their respective catchment areas. To evaluate the consequences of non-response, the population that is not included in the study can be analyzed via the clinic's data register where it should be clear whether the patient participated in the study or did not.

Research plan

Randomized double-blind study including 1400 patients meeting the inclusion criteria. Half of patients will receive glutamate infusion (1.65 ml/kg per hour of 0.125 M L-glutamate solution) which is started when the aorta is cross-clamped and the heart arrested. The infusion is then given for 2 hours after removal of the cross-clamp and start of reperfusion. Then another 50 ml of the solution is given with the infusion rate reduced by half. A maximum of 500 ml of solution is given. If the infusion volume is insufficient due to high body weight or long aortic cross-clamp time, the last 50 ml are given at halved infusion rate. The other patients receive infusion of physiological saline according to the corresponding schedule.

Specification of endpoints

As many leading medical journals require adjustment for multiple comparisons, the study will only have one primary endpoint based on the experience from the first GLUTAMICS study.

Primary endpoint:

The primary endpoint is a composite of a) cardiac mortality (30-days + hospital mortality) b) significant postoperative heart failure according to pre-specified criteria c) postoperative renal failure
.

The criteria for significant postoperative heart failure are postoperative heart failure with NT-proBNP > 5000 ng/l on the third postoperative day requiring ≥ 3 days of intensive care with persistent inotropic treatment 48 hours after surgery and at least 24 hours of total treatment time in prespecified doses (please refer to Investigator's Guide). In exceptional cases, a blinded Clinical Endpoints Committee assesses whether significant postoperative heart failure has existed (see Non-response).

Cardiac mortality is defined as deaths where the initiating cause was cardiac. Cardiac mortality is decided by a blinded Clinical Endpoints Committee.

Postoperative renal failure is defined as new dialysis requirements or duplication of p-creatinine.

Secondary endpoints and substudies

Secondary endpoints are not planned for the above reasons, but important clinical variables will be analyzed and reported.

Smaller substudies may become relevant and will be specified and reported to the Medical Products Agency and ClinicalTrials.gov before they are started.

Safety endpoints

Postoperative mortality (30 days + hospital), stroke within 24 hours and SUSAR.

Sample size determination and statistics

The sample size is based on available results from the first GLUTAMICS study. The power calculations have been done in consultation with statistical experts (80% power; 5% risk level; two-sided test; 50% reduction of an "event" occurring in 7% gives rounded 650 patients/group. The number of patients has then been rounded up to 1400 to compensate for any missed sampling and other dropouts.

Interim analysis will be performed by external independent statisticians after 800 patients in a way that does not increase the statistical requirements regarding the primary endpoint. An adaptive design using a surrogate variable (cardiac mortality + significant postoperative heart failure) will be used, which is considered to correlate with the primary endpoint and which will not be reported as a result. Although statistical significance should exist at interim analysis, the study will continue until a minimum of 1000 patients have been included. Please see stop criteria.

Categorical variables will be analyzed with two-sided Chi-2 except when the expected cell frequency is below 5 in which case Fisher's exact test will be applied.

Continuous variables will be analyzed with two-tailed t-test or Mann-Whitney U test depending on the distribution of the data.

Incomplete data and dropouts

In accordance with the exclusion criteria, the patient may be excluded from the study in the event of unforeseen significant expansion of the surgical procedure that greatly overshadows the dignity of the planned operation (e.g. aortic ascending surgery, severe bleeding complications intraoperatively). In addition, the patients are analyzed according to intention to treat.

The non-response rate is estimated to be less than 1%. In the first Glutamics study, the dropout rate was 4 patients out of 865 who accepted participation in the study. The non-response rate will be presented when the study is reproted.

Missed sampling (NT-proBNP) on day 3 will warrant sampling the following day. If this is also missed and the patient meets the criteria for intensive care and inotropic therapy for significant postoperative heart failure, an assessment is made by a blinded Clinical Endpoints Committee as to whether significant postoperative heart failure has occurred.

Timetable

Grants will be applied for from VR and the Swedish Heart-Lung Foundation in spring 2013. This requires approval from EPN (Ethical Review Board) and the Medical Products Agency. The EPN application was submitted on December 22, 2011. If funding can be obtained, we expect to be able to start in the spring of 2013 at several Swedish cardiac surgery centers. All patients are expected to be recruited within 2 - 3 years.

Patient information and informed consent

All patients who meet the inclusion criteria for the study receive oral and written information about the study. Written consent is obtained where the patient also consents to any future quality control by independent reviewers.

Ethical considerations

Postoperative heart failure remains the leading cause of prolonged intensive care, multi-organ failure and death after heart surgery. The risk is particularly high if the patient is operated on due to unstable angina with threatening myocardial ischemia (lack of oxygen) or if the patient already has impaired heart function before surgery. Currentl evidence suggests that glutamate stimulates the natural recovery of the heart after ischemia (lack of oxygen), which should be more gentle than using conventional inotropic drugs to stimulate contractility regardless of whether the heart has recovered or not. The potential benefits of the study are therefore considered to be large.

In addition to the infusion of 300 - 500 ml glutamate solution or saline, patients will receive normal care.

Glutamate is an endogenous amino acid and one of the most abundant free amino acids in heart muscle cells. In connection with ischemia, glutamate is consumed, and the heart attempts to replace this by extracting glutamate from the blood. The extraction rate is about 50%. Glutamate levels rise rapidly with oral and intravenous administration. In isolated individuals, transient discomfort (Chinese restaurant syndrome: flush, pain sensations in the face-head, chest tightness) has been reported. However, in the first GLUTAMICS study, no adverse reactions related to glutamate were observed.

Despite very extensive use of glutamate, there is no evidence that exogenous administration caused nerve damage or other lasting complications in humans or primates. One reason for this may be that glutamate does not cross the blood-brain barrier. In addition, glutamate levels in the central nervous system are up to 50 times higher than in blood. The glutamate infusion that patients receive increases whole blood levels 2-3 times, which has been shown to double the myocardial uptake of glutamate. Higher infusion rates have not been shown to result in an additional increase in glutamate uptake.

Clinical significance and clinical experience: Experience from the first GLUTAMICS study suggests that glutamate could halve the risk of developing severe heart failure and thus also cardiac mortality. According to O'Connor, postoperative heart failure was the primary cause of 2/3 of the deaths after heart surgery in Northern New England and the single most important factor that distinguished clinics with good and worse results.

Our group has been using glutamate in clinical practice for 20 years in Linköping and experience so far shows a mortality that has been 75% lower than expected according to EuroSCORE in coronary artery surgery. Compared to national and international results, we have noted that the risk of renal failure (which is considered the most sensitive indicator of the quality of heart failure treatment) after coronary artery surgery has been at least 5 times lower (regardless of which outcome variable you look at).

Patients admitted to surgery with severely impaired left ventricular function is the cohort where we have previously used glutamate for prevention to the greatest extent. In a recently published study involving more than a hundred patients, we recorded a mortality of 1.0% in a cohort where the expected risk-corrected mortality according to logistic Euroscore was 8.3%. Since these types of results are influenced by other factors, they must be interpreted cautiously, but they are consistent with the results from the first GLUTAMICS study.

The biochemical properties of glutamate mean that the clinical effects that we want to confirm in GLUTAMICS II should also be reproducible in studies involving other patient groups with acute ischemic heart disease. The major future clinical significance may therefore be found in the field of cardiological treatment of acute myocardial infarction and other conditions with acute myocardial ischemia.

Overall risk-benefit evaluation

Glutamate has metabolic properties that may increase tolerance to myocardial ischemia and improve recovery of cardiac metabolism and function after ischemia. These properties are desirable in cases when conventional pharmacological treatment and myocardial protection are insufficient. The need for alternative treatment is reinforced by the fact that traditional treatment of postoperative heart failure with inotropic drugs increases myocardial oxygen demand disproportionately in relation to effect and thus may aggravate the underlying myocardial damage. This may explain why postoperative heart failure has been identified as the dominant cause of fatal outcome after coronary artery surgery. In the first GLUTAMICS study, we saw a relative risk reduction in excess of 50% in most high-risk groups (see Table 1) to develop heart failure leading to prolonged intensive care or death.

Patients with unstable angina (CCS class IV) currently constitute the numerically largest risk group for the development of postoperative heart failure after heart surgery. Patients admitted to surgery with impaired heart function are also an important high-risk group as they have poorer margins to withstand an ischemic injury.

Against this should be set potential side effects reported below. However, the risks of side effects, triggering Chinese restaurant-like disorders, asthma and urticaria with the current doses of glutamate are considered very small and will be further limited by excluding patients with ambiguous food allergy and asthma. In connection with the clinical use of glutamate for twenty years and in the first GLUTAMICS study, we have not seen such side effects. Against the above background, we believe that the potential benefits for patients are larger than the risks. Please see the Investigator's Manual for more details and references.

Administration of glutamate

Glutamate levels rise rapidly with both oral and intravenous administration. In connection with cardiac surgery, glutamate has been administered via cardioplegia solution (additive) or intravenously. There are experimental data suggesting that the transport of glutamate across the cell membrane is inhibited by cardioplegia (hyperkalemic solution) and that the cardiomyocytes instead leak glutamate during cardioplegic arrest. The main reason for the choice of intravenous administration in the current study is to stimulate the post-ischemic recovery of the heart during the first hours after surgery.

Dosage of glutamate

Available data suggest that cardiac uptake of glutamate is strongly correlated to arterial levels in patients undergoing coronary artery surgery. It is also known that myocardial uptake of glutamate increases when plasma levels are increased by intravenous administration. However, an increase in arterial levels (whole blood) more than 2-3 times has not been found to produce any further increase in the heart's glutamate uptake. These arterial levels are achieved with an infusion of 1.65 ml/h and kg body weight of a 0.125 M glutamic acid solution.

The total infusion volume according to the planned infusion schedule (see under research plan) will be as follows depending on the patient's weight: 70 kg – 368 ml; 80 kg – 413 ml; 90 kg 458 ml; 100 kg – 500 ml. The calculations assume an average aortic cross-clamp time of 45 minutes. The maximum amount infused is 500 ml.

Details regarding the composition of the solution can be found in pharmaceutical documentation.

Discontinuation of infusion

The infusion should be stopped if signs of allergic reaction (rash, bronchial obstruction, anaphylaxis) or SUSAR occur. The infusion is also discontinued if the exclusion criterion "significant extension of the procedure that overshadows the planned operation" is met, such as aortic dissection or aortic aneurysm requiring replacement of the ascending aorta, need for valve replacement or complicated intra-operative surgical bleeding with repeated circulatory effects.

Medication management

Medication management is conducted according to recommendations from the manufacturer Apoteksbolaget.

Randomization, control treatment, patient identification

Randomization in varying block sizes is conducted externally at Apoteksbolaget in Umeå, where the preparation of the solutions also takes place.

The randomization code is on the infusion bottle as a self-adhesive label that is removed and pasted into the patient's CRF when the infusion is started. This is the stage when the patient is formally included in the trial.

The masking is double-blind. In the study, placebo consists of physiological saline. Both the glutamate solution and placebo are clear transparent solutions that are indistinguishable to the eye. The bottles are marked with a patient code that is registered in CRF and the clinic's data register. Patient identification lists will be kept allowing identification of patients during data quality control.

The randomization code will be stored at Apoteksbolaget in Umeå. It should be possible to break this for individual patients if side effects are suspected. This is done by the principal responsible for the preparation of solutions/randomization at the request of the investigator or by a delegated person.

Safety monitoring

All patients are continuously monitored the first day with invasive hemodynamic monitoring (CVP, arterial pressure), ECG, diuresis, level of consciousness, control of drainage losses. In addition, patients with hemodynamic instability are monitored with PA catheter and SvO2 measurements. Transesophageal echocardiography is routinely performed peroperatively and, if necessary, postoperatively in case of hemodynamic instability. After the first day, the degree of monitoring is adjusted depending on the patient's condition. Early mobilization is routine.

Neurology status is checked after awakening, and brain scan (CT) is performed in case of suspected neurological damage. Biomarkers for myocardial and kidney damage markers are checked on the first (CKMB, p-creatinine) and third postoperative day (troponin-T, p-creatinine). Biomarkers for heart failure markers are checked preoperatively and on the third postoperative day (NT-proBNP).

Side effects and toxicology

Plasma glutamate levels rise rapidly with both oral and intravenous administration Despite a very extensive use of this substance, primarily as a flavoring substance but also in infusion solutions, there is currently no documentation showing that exogenously administered glutamate caused permanent damage in humans or primates.

Conventional toxicity studies in several species have not provided any indications for carcinogenic or teratogenic effects. No effect on reproduction has been demonstrated either.

In the CNS, glutamate is the dominant neurotransmitter. Under certain conditions, glutamate is considered to act as an excitotoxin and contribute to processes leading to neuronal damage or neuron death in acute and chronic neurological diseases. Glutamate is considered to be involved in the pathogenesis of ischemic brain injuries and research today is working to find glutamate receptor antagonists that could limit the damage.

Damage to the CNS has been induced in certain animal species, especially rodents, with large doses of exogenously administered glutamate. Despite very extensive use of glutamate, there is no evidence that exogenous administration of glutamate caused permanent nerve damage in humans or other primates. One explanation is that glutamate in primates does not cross the blood-brain barrier or placenta.

Theoretically, extracorporeal circulation with use of the heart-lung machine could affect the permeability of the CNS. However, glutamate has in some parts of the world been routinely used in connection with cardiac surgery as an additive in cardioplegia solutions (resulting in plasma levels that are significantly higher than those aimed for in our study) without finding any evidence of an increased risk of CNS complications. In our own clinical practice, glutamate infusion has been used as metabolic support in the treatment of postoperative heart failure. A multivariable analysis of CNS complications after cardiac surgery did not show any tendency to increase the risk of CNS complications, but rather a tendency towards the reverse relationship.

In a neurotrauma cohort, a conventional amino acid solution (glutamate 3.75 grams/L) was found to increase plasma glutamate twice, but no difference in outcome was found between a group of patients who received amino acids versus the group that did not receive amino acids.

In this context, it may be worth considering that glutamate levels in the central nervous system are about 50 times higher than in blood. The glutamate infusion that we intend to give increases blood levels 2-3 times.

In the first GLUTAMICS study involving 861 patients, the risk of neurological adverse events was evaluated with close monitoring of neurological events but also by sampling of the biomarker S-100B in a subpopulation. No differences or trends were found between the glutamate and control groups regarding clinical neurological events (in manuscript).

Although glutamic acid is an endogenous amino acid, side effects and allergic reactions to glutamatsaltes have been discussed. There are three conditions in particular that have attracted interest in the medical literature. This applies to the "Chinese restaurant syndrome", glutamate-induced asthma and glutamate-induced urticaria. The most noted of these conditions is the so-called "Chinese restaurant syndrome" (flush, pain sensations in the face-head, tightness in the chest). Several blinded studies, some conducted on people who reported increased sensitivity to glutamate, have failed to confirm the link to glutamate as the study groups had the same amount of symptoms regardless of whether they received glutamate or not. Similar results have been obtained in suspected glutamate-induced asthma. However, there are studies that suggest that glutamate can trigger Chinese restaurant syndrome, asthma and urticaria in predisposed individuals.

Although there have been reasons to question the methodology in some of the studies, asthma and history of food allergy that rises suspicion of hypersensitivity to glutamate will constitute exclusion criteria in the planned study.

For more details and references regarding adverse reactions and toxicology, please refer to the IB.

Adverse events (AE)

Adversal events (AE) are common after cardiac surgery and all patients suffer from some degree of postoperative pain, bleeding and fever. Serious adverse events (SAE) are defined as AEs that have been life-threatening or fatal or that have led to prolonged hospital care or permanent harm. SAE is also common after heart surgery. Atrial fibrillation, which is the most common cause of prolonged care, is seen in a third of patients.

In Glutamics II, AE will be registered on a special form and divided into minor AE and SAE. Minor AEs are those that do not meet the criteria for SAE. To determine whether the study drug had any effect on AE, AE in the control group and treatment group will be compared statistically. AE will be reported structured according to organs affected: CNS, heart-circulation, lungs, gastrointestinal tract, kidneys, skin. Infections are reported according to the wound or organ system affected. Postoperative nausea is reported if it prompted antiemetic treatment.

Reporting of adverse events, adverse reactions and SUSAR

Serious adverse events (SAE) and SUSAR are reported on a special fax form (copy attached) to the sponsor within one day of participating investigators becoming aware of the incident. Code breaking rules are specified in the attached investigator's manual.

Suspicion of side effects and SUSAR will lead to a report to the Medical Products Agency in accordance with the regulations issued by the Medical Products Agency. The sponsor is

responsible for this and reports of SUSAR that have been life-threatening or fatal are reported within 7 days and others within 15 days.

Suspicion of side effects during the study will also be reported to the local ethics committee.

The sponsor makes an annual summary of AE to the Medical Products Agency.

Monitoring, quality control and interim analyses

External professional monitoring according to GCP standard including monitoring of sponsor, investigator, trial location, pharmacy, handling of infusion solutions, source data control, SAE and SUSAR reporting.

Internally, the study will be monitored by weekly responsible employees to continuously ensure an adequate inclusion procedure, complete sampling, archive and check registered CRFs and to register dropouts.

External statistical expertise from Linköping University will perform interim analysis, which is planned to take place after 800 patients have been included (see below).

The Medical Products Agency will be given the opportunity to carry out/order an independent inspection.

Clinical Endpoints committee

The study will have a clinical endpoints committee consisting of consultants in cardiothoracic surgery and crdiothoracic anesthesia from the participating centers. The Committee will blindly assess all cases that died and determine whether the cause was cardiac or initiated by cardiac causes. In exceptional cases, the Committee decides whether postoperative heart failure has occurred (see Incomplete data and dropouts).

Reasons for early termination of the study

If monitoring or interim analysis would provide evidence or suspicion of an increased risk of stroke or an adverse effect on mortality, the study will be terminated prematurely. Should continuous monitoring provide evidence of unexpected and serious adverse reactions beyond those stated above, the study will also be discontinued. Safety aspects will thus be prioritized and the trial can be terminated prematurely before the occurrence of any side effects becomes statistically significant. In a situation where the next event may involve a p-value <0.10 regarding negative effect on stroke or mortality, contact LV will be made to discuss the issue. Sample size will be corrected after interim analysis. If a positive treatment effect regarding the surrogate endpoint is found already at the interim analysis, the study will still continue until a minimum of 1000 patients have been included.

The study will be discontinued if there is no absolute difference in surrogate endpoint (cardiac mortality + significant heart failure) in favour of the treatment group at interim analysis. While interim analysis is ongoing, patients will be included.

Insurance

Heart surgery carries risks of complications, pain and suffering. It is very unlikely that the study would have a negative impact on this. Insurance coverage is therefore provided by the existing patient insurance.

Information of staff

All categories of staff concerned will be informed orally about the study, its background and purpose. Contact persons will be appointed who will continuously answer questions that arise and in addition, written information about the study will be well available at all affected care units.

Sponsor

Rolf Svedjeholm, Thoraxkir sektionen, Thorax-kärlkliniken, US, Linköping

Investigators

Rolf Svedjeholm, överläkare, Thoraxkir sektionen, Thorax-kärlkliniken, US, Linköping
Erik Håkansson, överläkare, klinikchef Thoraxanestesi, Thorax-kärlkliniken, US, Linköping
Farkas Vanky, överläkare, Thoraxkir sektionen, Thorax-kärlkliniken, US, Linköping

Örjan Friberg, överläkare, Thoraxkir sektionen, Thoraxkliniken, US, Örebro
Mårten Vidlund, specialistläkare, Thoraxkir sektionen, Thoraxkliniken, US, Örebro

Lars Algotsson, överläkare, Kliniken för thoraxkirurgi, anestesi och intensivvård, Skånes Universitetssjukhus, Lund.

Investigators from other Swedish cardiac surgery centers have expressed interest and will be recruited if and when funding for the study is obtained. Please see timetable.

Allocation of responsibilities and applicable delegations within the trial group

The principal investigator is responsible for delegating tasks within the trial to different employees.

Reporting and publishing

The results of the study will be made available regardless of outcome and submitted for peer-review to internationally leading journals in the field of circulation / cardiology / cardiac surgery. The choice of journal will be discussed within the trial group when the results are ready. Advance reporting of the results may be relevant at international congresses in the field of circulation.

Archiving of trial materials

Primary data will be archived and available for independent external monitoring for 15 years. Archiving will take place in such a way that patient confidentiality and integrity are not compromised. CRFs will be kept locked and CRFs shall not be able to allow identification of individual patients other than with access to patient code and identification list stored in separate locked areas.

References

1. Svensson SE, Svedjeholm R, Ekroth R, Milocco I, Nilsson F, Sabel K G, William-Olsson G. Trauma metabolism and the heart; uptake of substrates and effects of insulin early after cardiac surgery. J Thorac Cardiovasc Surg 1990; 99: 1063 - 1073.
2. Svedjeholm R, Svensson SE, Ekroth R, Milocco I, Nilsson F, Vinnars E, Wernerman J. Trauma metabolism and the heart; studies of heart and leg amino acid flux after cardiac surgery. Thorac Cardiovasc Surgeon. 1990; 38: 1-5.
3. Svedjeholm R, Ekroth R, Joachimsson PO, Ronquist G, Svensson SE, Tydén H. Myocardial uptake of amino acids and other substrates in relation to myocardial oxygen consumption four hours after cardiac surgery. J Thorac Cardiovasc Surg 1991; 101: 688 - 694.
4. Svedjeholm R, Ekroth R, Joachimsson PO, Tydén H. High dose insulin improves the efficacy of dopamine early after cardiac surgery. A study of myocardial performance and oxygen consumption. Scand J Thorac Cardiovasc Surg. 1991; 25: 215 - 221.
5. Svedjeholm R, Hallhagen S, Ekroth R, Joachimsson PO, Ronquist G. Dopamine and High-dose Insulin infusion (Glucose-Insulin-Potassium) after a Cardiac Operation. Effects on Myocardial Metabolism. Ann Thorac Surg 1991; 51: 262 - 270.
6. Svedjeholm R, Huljebrant I, Håkanson E, Vanhanen I. The Rationale for Metabolic Support with Amino Acids and GIK (Glucose-Insulin-Potassium) in Cardiac Surgery. Ann Thorac Surg.1995; 59:S15-22.
7. Svedjeholm R, Huljebrant I, Håkanson E, Vanhanen I. Glutamate and high-dose glucose-insulin-potassium (GIK) in the treatment of severe cardiac failure after cardiac operations. Ann Thorac Surg. 1995; 59: S23-30.
8. Svedjeholm R, Vanhanen I, Håkanson E, Joachimsson PO, Jorfeldt L, Nilsson L. Metabolic and hemodynamic effects of intravenous glutamate infusion early after coronary surgery. J.Thorac.Cardiovasc.Surg. 112(6):1468-1477, 1996.
9. Vanhanen I, Svedjeholm R, Håkanson E, Joachimsson PO, Jorfeldt L, Nilsson L, Vanky F. Assessment of myocardial glutamate requirements early after coronary artery bypass surgery. Scand Cardiovasc J 1998; 32: 145-152.
10. Vanhanen I, Håkanson E, Jorfeldt L, Svedjeholm R. Myocardial uptake and release of substrates in patients operated for unstable angina: impact of glutamate infusion. Scand Cardiovasc J 2003; 37: 113-120.

11. Rolf Svedjeholm, Mårten Vidlund, Ingemar Vanhanen, Erik Håkanson. A metabolic protective strategy could improve long-term survival in patients with LV-dysfunction undergoing CABG. Scand Cardiovasc J 2010; 44: 45-58.
12. Mårten Vidlund, Jonas Holm, Erik Håkanson, Örjan Friberg, Lena Sunnermalm, Farkas Vanky, Rolf Svedjeholm. The S-100B substudy of the GLUTAMICS-trial: Glutamate infusion not associated with sustained elevation of plasma S-100B after coronary surgery. Clin Nutr 2010; 29: 358-64.
13. Mårten Vidlund, Erik Håkanson, Örjan Friberg, Sören Juhl-Andersen, Jonas Holm, Farkas Vanky, Lena Sunnermalm, Jan-Olov Borg, Rajiv Sharma, Rolf Svedjeholm. GLUTAMICS – a randomized clinical trial on glutamate infusion in patients operated for acute coronary syndrome. American College of Cardiology, New Orleans, April 2-5, 2011. (JACC Volume: 57 Issue: 14 Supplement: 1 Pages: E938-E938 Published: APR 5 2011)
14. Mårten Vidlund: Glutamate for metabolic intervention in coronary surgery – with special reference to the GLUTAMICS-trial. Thesis 2011: Örebro Studies of medicine 58.

Table 1

	**Number Control/ Glutamate**	**Control**	**Glutamate**	**RR**	**95% CI**	**p-value**

Isolated CABG	413/411	16 (3.9%)	6 (1.5%)	0.38	0.15-0.95	0.04
NSTEMI only	68/59	1 (1.5%)	1 (1.7%)	1.15	0.07-18	0.92
Diabetics	108/98	4 (3.7%)	4 (4.1%)	1.10	0.28-4.3	0.89
Non-diabetics	305/313	12 (3.9%)	2 (0.6%)	0.16	0.04-0.72	0.02
CSS class IV	233/225	16 (6.9%)	3 (1.3%)	0.19	0.06-0.66	0.008
Left main stenosis >50%	141/174	10 (7.1%)	1 (0.6%)	0.08	0.01-0.63	0.02
Female gender	77/70	7 (8.5%)	2 (2.7%)	0.31	0.26-1.2	0.14
EuroSCORE≥8	63/83	9 (14.3%)	4 (4.8%)	0.34	0.11-1.05	0.06
Severe LV-dysfunction	20/14	5 (25%)	1 (7.1%)	0.29	0.04-2.2	0.23
Emergency*	13/14	4 (31%)	1 (7.1%)	0.23	0.03-1.6	0.16
LV-failure at weaning	14/18	8 (57%)	2 (11%)	0.19	0.05-0.78	0.02

Table 1. The incidence of severe postoperative circulatory failure in the control group and glutamate group in different subgroups undergoing isolated CABG. The relative risk ± 95% confidence interval of developing severe postoperative circulatory failure associated with glutamate treatment. Results are approximative given the small number of events. * Emergency according to EuroSCORE definition. RR= relative risk; CI= confidence interval.

Table2. Outcome data for patients with LV-failure at weaning from CPB (median+ interquartile range, mean±SD or count n %).

	Placebo n=18	*Glutamate n=20*	*p-value*
a-Lactate mmol/L 5 minutes after	2.5±1.0	1.7±0.5	*0.004*

protamine			
Hemodynamic state at completion of surgery			
-Unstable with inotropes / IABP	31% (5/16)	0	*0.01*
Severe circulatory failure	56%(10/18)	15%(3/20)	*0.02*
Postop increase of p-Creatinine (Δ µmol/L)	58±87	19±34	0.08
Renal injury (increased creatinine x2)	22%	0%	*0.03*
Ventilator treatment (hours)	7.4 [5.8-49]	5.0 [3.3-8.0]	*0.02*
ICU stay (hours)	92 [41-139]	25 [18-57]	*0.02*

AMENDMENT 1

Linköping 2015-09-10

Läkemedelsverket
Kliniska prövningar & Licenser

Box 26
751 03 Uppsala
(Swedish Medical Product Agency)

AMENDMENT concerning previously approved clinical trials (Dnr151:2011/96689) "GLUTAMICS II: Metabolic intervention with glutamate in surgery for ischemic heart disease" (EudraCT number: 2011-006241-15)

For economic reasons, the above study has not yet started. We have now received SEK 1.2 million in grants from the Swedish Heart-Lung Foundation for the GLUTAMICS II study. However, we have not received any funding from VR and the funding will thus be insufficient for the originally planned study.

The Swedish Heart-Lung Foundation has approved that we use their grant for a simplified version of GLUTAMICS II, which prompts this amendment.

GLUTAMICS II will be a study with a simpler design that requires fewer patients and carries lower cost. For economic reasons (n=400) all infusion solutions are manufactured by APL in one batch with a 24-month shelf life, which means that all patients should be included within a two-year period. Should interim analysis show that more patients than the currently planned 310 are needed and the number exceeds 400, new grant applications will be made.

As before, the goal is to show that intravenous glutamate infusion reduces the risk of postoperative heart failure after coronary artery surgery. Instead of a dichotomous clinical outcome variable "severe heart failure yes/no", we use NT-proBNP as a biomarker heart failure marker postoperatively.

Today, there are no generally accepted criteria for heart failure after heart surgery. The first GLUTAMICS trial was unique in that it had a blinded endpoints committee that relied on strict pre-specified criteria for heart failure previously evaluated against long-term survival. NT-proBNP was simultaneously evaluated blindly against these pre-specified criteria. NT-proBNP was found to be a strong predictor of severe postoperative heart failure. This could be a breakthrough for the evaluation of heart failure treatment in association with heart surgery. These data will be published and will be summarized in a thesis by Huiqi Jiang, who was recently registered as a PhD student at Linköping University (see attached research program). The advantage of this endpoint is the need for fewer patients and that the primary endpoint can be reproduced in other future studies.
Intravenous glutamate infusion reduced the postoperative increase in NT-proBNP in all risk groups including diabetics (where we clinically saw no effect in the first study) in the first study.

In the new simplified version of GLUTAMICS II, we intend to show that intravenous glutamate infusion reduces the rise in NT-proBNP from preoperative level to postoperative level on day 3. Secondary endpoints are the absolute levels of NT-proBNP on the first and third postoperative days.

We have broadened the inclusion criteria somewhat, but still focus on patients at risk who need coronary artery surgery. Patients undergoing bypass of at least two-vessel diseaase with or without other concomitant procedures and preoperatively assessed as having at least moderate to high risk of postoperative mortality (EuroSCORE II ≥ 3.0) are now candidates for inclusion in the study. The sample-size calculations have been made by an external statisticians based on data from patients with EuroSCORE II ≥ 3.0 from the first GLUTAMICS trial.

Since we also include CABG + valve procedures in the study, this means that some patients have longer aortic cross-clamp times. In order for the infusion solution to last the planned two hours and 30 minutes after reperfusion, the infusion is started a little later, 10-20 minutes before anticipated removal the aortic cross-clamp. The total infusion volume will thus be slightly smaller in most patients.

Due to the modification of the inclusion criteria, the exclusion criteria are also affected.

The following changes in the study protocol have been made:

Inclusion criterion: EuroSCORE II ≥ 3.0 and bypass of minimum two-vessel disease with or without other procedure.

Exclusion criteria: diabetes, other concomitant procedure with some exceptions are deleted. Please see protocol.

Randomization: Randomization is stratified for patients undergoing isolated coronary artery surgery and for those undergoing CABG + concomitant procedure.

Primary endpoint: elevation of NT-proBNP from the day before surgery to the third postoperative day

Secondary endpoints: absolute levels of NT-proBNP on the first and third postoperative days

Substudy – subgroup analysis: blood is saved for evaluation of future biomarkers for heart failure and myocardial injury. Patients who undergo isolated CABG or CABG with concomitant procedure are also analyzed separately.

Sample size: Number of patients n=310 (previously 1400). Interim analysis after 160.

Timeline: study completed within 18-24 months

Start of infusion: 10-20 minutes before reperfusion (removal of the aortic force)

Infusion volume: slightly smaller, see protocol

Clinical endpoints committee: not required

Monitoring: simplified, budget-adapted, please see trial protocol

New investigator in Linköping: Jonas Holm

New participating centres + investigators: Umeå – Anders Holmgren, Karlskrona – Andreas Borsiin.

To avoid delaying the start of the trial, amendments are sent now before we have finalized participating centers. Sahlgrenska University Hospital will probably decide to participate next week. Another investigator from Örebro will be added. We currently have two new centers (Umeå and Karlskrona). The formal paperwork only included space for a new center. According to an email from Katarina Thor at the Medical Product Agency, it should be sufficient to register new centers to EPN (Ethical review Board). I will therefore put the registration to the Regional EPN (Ethical review Board) in Linköping on hold until we have completed the CVs and resource certificates from all centers and investigators involved in the trial.

Sincerely

Rolf Svedjeholm
Professor, överläkare
Thorax-kärlkliniken
Universitetssjukhuset
581 85 Linköping

Tel: 010-1034825
Fax: 013-100246
Mobil: 0706-043052
E-post: rolf.svedjeholm@regionostergotland.se

PS I enclose the application form, trial protocol with and without marked changes (yellow marked = new, red marked in brackets = removed) and Huiqi Jiang's PhD project referred to in this letter. If any other documents are required, I would be grateful for a reply soon to avoid delays in the processing.

AMENDMENT 2

Linköping 2015-11-17

Läkemedelsverket
Kliniska prövningar & Licenser

Box 26
751 03 Uppsala

(Swedish Medical Product Agency)

AMENDMENT 2 concerning previously approved clinical trials (Dnr 5,1-2015-77379) "GLUTAMICS II: Metabolic intervention with glutamate in surgery for ischemic heart disease" (EudraCT number:2011-006241-15)

As we started looking for suitable patients to include, we have noticed that some patients with poor left ventricular function who would be particularly suitable for the trial do not reach EuroSCORE II ≥ 3.0. We also have observed that some patients can be included only due to advanced age and history of extracardiac atherosclerotic complications without having risk factors for postoperative heart failure. For this reason, the inclusion criteria have been slightly modified as follows.

With the modified criteria, we can include patients with poor left ventricular function that we would previously miss. In addition, the specification of the EuroSCORE II criterion helps ensure that the patients included have at least one cardiac factor of significance. The risk of including patients with high EuroSCORE II mainly due to stroke risk or other reasons where the intervention cannot be expected to have an impact is thereby reduced.

The sample-size calculation based on data from the first Glutamics study is not changed by the new criteria.

We are also adding a new centre with investigators approved by the Ethical Review Board EPN (Dnr 2015/33332).

The following changes in the study protocol are reported:

Inclusion criteria:

Patients accepted for bypass surgery of at least two-vessel disease or left mainstem stenosis with or without concomitant procedure and who have either:

- EuroSCORE II ≥ 3.0 and where at least one of the following factors contributes
- LVEF < 0.50
- CCS Class IV
- AMI < 90 days

- Emergent / semi-emergentsurgery (emergency / urgent)
- CABG + aortic or mitral valve

Poor left ventricular function (LVEF≤ 0.30) regardless of EuroSCORE II.

The operation should be performed with a heart-lung machine

New participating center + investigator: Gothenburg - Mårten Vidlund. Approved by Ethical Review Board EPN (Dnr 2015/333-32)

Sincerely,

Rolf Svedjeholm
Professor, överläkare
Thorax-kärlkliniken
Universitetssjukhuset
581 85 Linköping

Tel: 010-1034825
Fax: 013-100246
Mobil: 0706-043052
E-post: rolf.svedjeholm@regionostergotland.se

PS I attach the registration form, trial protocol with and without marked changes (yellow marked = new)

AMENDMENT 3

Linköping 2016-08-01

Läkemedelsverket
Kliniska prövningar & Licenser

Box 26
751 03 Uppsala
(Swedish Medical Product Agency)

AMENDMENT 3 concerning previously approved clinical trial (Dnr 5.1-2015-96404) "GLUTAMICS II: Metabolic intervention with glutamate in surgery for ischemic heart disease" (EudraCT number: 2011-006241-15)

Preoperative treatment with inotropic drugs is an exclusion criterion that existed in the first Glutamics study and hence was transferred to the second study. During the course of the study, however, we have noted that a number of patients who would have been suitable for the study could not be included as a routine to give preventive Levosimendan infusion began to be used by individual colleagues on patients with preoperatively poor heart function. In the first Glutamics study, post-hoc analysis showed that patients receiving inotropic drugs were the ones who seemed to benefit most from glutamate infusion. We will therefore remove treatment with inotropic drugs preoperatively as an exclusion criterion.

The following changes are notified in view of the above background:

New participating centres + investigators: Uppsala – Lena Jideus. Application to the Ethical Review Board EPN will be sent.

New investigator in Örebro: Gabriele Ferrari. Application to the Ethical Review Board EPN will be sent.

Exclusion Criteria: Treatment with inotropic drugs preoperatively is removed as an exclusion criterion. Please see protocol. Application to the Ethical Review Board EPN will be sent.

Sincerely,

Rolf Svedjeholm
Professor, överläkare
Thorax-kärlkliniken
Universitetssjukhuset
581 85 Linköping

Tel: 010-1034825
Fax: 013-100246
E-post: rolf.svedjeholm@regionostergotland.se

PS I attach the application form, trial protocol with and without marked changes (yellow marked = new).

AMENDMENT 4

Linköping 2017-09-21

Läkemedelsverket
Kliniska prövningar & Licenser
Box 26
751 03 Uppsala
(Swedish Medical Product Agency)

AMENDMENT 4 concerning previously approved clinical trial (Dnr 5.1-2015-96404) "GLUTAMICS II: Metabolic intervention with glutamate in surgery for ischemic heart disease" (EudraCT number: 2011-006241-15)

The study has been running since November 2015 and the rate of inclusion has been somewhat lower than expected. To date, more than 150 patients have been included. Interim analysis is planned for 160 patients and is expected to take place in 6 weeks. For several reasons, it is important to avoid interruption of the study and this amendment is because the shelf-life date for the infusion solutions expires 2017-09-30.

The following change is reported in view of the above:

The manufacturer of the infusion solutions APL has, after verification, assessed that the shelf life can be extended from 24 months to 36 months for the current batch, meaning a new expiration date 2018-09, provided that the Medical Products Agency gives its consent. See attached updated Certificate of Compliance.

After approval of the extended shelf life, relabeling with a new expiry date must be done by an authorized person in hospital pharmacies and at APL for undistributed solutions. The solutions will be pharmaceutically checked again at the new expiration date.

Sincerely,

Rolf Svedjeholm
Professor, överläkare /Sponsor och huvudprövare GLUTAMICS II
Thorax-kärlkliniken
Universitetssjukhuset
581 85 Linköping

Tel: 010-1034825
Fax: 013-100246
Mobil: 0706-043052
E-post: rolf.svedjeholm@regionostergotland.se

PS I attach copies of the Certificate of Compliance and APL's license to manufacture pharmaceuticals.

STATISTICAL ANALYSIS PLAN

The sample size is based on available results on NT-proBNP from the first GLUTAMICS-trial. In that study the following increase of NT-proBNP was observed in patients undergoing CABG or CABG with concomitant procedure who fulfilled inclusion criteria for the planned trial from preoperative values to postoperative day 3 (mean ± Standard Deviation)

Glutamate (n=71): 5261 ± 4409

Placebo (n=62): 7112 ± 6454

Sample size estimation by statistical expertise (80% power, 5% risk level; two-sided test) suggests 141 patients in each group. To compensate for possible missed sampling and other causes for loss of data we plan to include a total of 310 patients.

Interim analysis will be performed by an independent external statistician after 160 patients in a way that does not increase the demands regarding statistical significance of the primary endpoints. An adaptive design implying that a surrogate variable (increase of NT-proBNP from preoperative level to postoperative day 1) known to correlate well with the primary endpoint will be used. Furthermore, regardless if statistical significance is reached the study will proceed until 310 patients have been included or until the expiry date of the study solutions. Stopping criteria are given in the Study protocol.

Continuous variables will analyzed with a two-tailed t-test or Mann-Whitney U test depending on the distribution of data.

Categorical variables will be analyzed with a two-tailed Chi square test unless expected cell counts are less than 5, then Fisher's exact test will be used.

Adjustment for extra-cardiac variables (age, gender, preoperative renal function, body mass index) known to influence NT-proBNP and preoperative cardiac function has been considered. However, we have decided to rely on this study as a pure randomized clincial trial without further statistical adjustment as it is based on a post-hoc analysis in a similar cohort and adequately powered.
