# Supplementary material for: Association of Glutamate Infusion With Risk of Acute Kidney Injury After Coronary Artery Bypass Surgery: A Pooled Analysis of 2 Randomized Clinical Trials
Source: JAMA Netw Open. 2024 Jan 22;7(1):e2351743. doi: 10.1001/jamanetworkopen.2023.51743 (PMC10804267; doi:10.1001/jamanetworkopen.2023.51743)
Supplement: Supplement 2. — Data Sharing Statement [file jamanetwopen-e2351743-s002.pdf]

Data Sharing Statement

Holm. Association of Glutamate Infusion With Risk of Acute Kidney Injury After Coronary Artery Bypass Surgery. *JAMA Netw Open*. Published January 22, 2024.
doi:10.1001/jamanetworkopen.2023.51743

Data
Data available: Yes
Data types: Deidentified participant data
How to access data: Deidentified data on reasonable request by e-mail to jonas.holm@liu.se or jonas.holm@regionostergotland.se
When available: With publication

Supporting Documents
Document types: Statistical/analytic code, Informed consent form
How to access documents: By e-mail request to jonas.holm@liu.se or jonas.holm@regionostergotland.se
When available: With publication

Additional Information
Who can access the data: Data described in the manuscript will be made available upon reasonable request for researchers whose proposed use has been approved and who meet the criteria for access to confidential data. Full study protocols for both trials are also available on request.
Types of analyses: Specified scientific purposes approved by an ethical review board
Mechanisms of data availability: With investigator support and a signed data access agreement
